# Deficiency of the mitochondrial ribosomal subunit, MRPL50, causes autosomal recessive syndromic premature ovarian insufficiency

**DOI:** 10.1007/s00439-023-02563-z

**Published:** 2023-05-06

**Authors:** Shabnam Bakhshalizadeh, Daniella H. Hock, Nicole A. Siddall, Brianna L. Kline, Rajini Sreenivasan, Katrina M. Bell, Franca Casagranda, Sadishkumar Kamalanathan, Jayaprakash Sahoo, Niya Narayanan, Dukhabandhu Naik, Varun Suryadevara, Alison G. Compton, Sumudu S. C. Amarasekera, Ridam Kapoor, Sylvie Jaillard, Andrea Simpson, Gorjana Robevska, Jocelyn van den Bergen, Svenja Pachernegg, Katie L. Ayers, David R. Thorburn, David A. Stroud, Gary R. Hime, Andrew H. Sinclair, Elena J. Tucker

**Affiliations:** 1 Murdoch Children’s Research Institute, Melbourne, Australia; 2 Department of Paediatrics, University of Melbourne, Melbourne, Australia; 3 Department of Biochemistry and Pharmacology, Bio21 Molecular Science and Biotechnology Institute, University of Melbourne, Parkville, Australia; 4 Department of Anatomy and Physiology, University of Melbourne, Parkville, Australia; 5 Department of Bioinformatics, Murdoch Children’s Research Institute, Melbourne, Australia; 6 Department of Endocrinology, Jawaharlal Institute of Postgraduate Medical Education and Research, Puducherry, 605006 India; 7 Victorian Clinical Genetics Services, Royal Children’s Hospital, Melbourne, Australia; 8 Univ Rennes, CHU Rennes, INSERM, EHESP, IRSET (Institut de Recherche en Santé, Environnement et Travail) – UMR_S 1085, 35000 Rennes, France; 9 CHU Rennes, Service de Cytogénétique et Biologie Cellulaire, 35033 Rennes, France; 10 School of Allied Health, College of Science, Health and Engineering, La Trobe University, Bundoora, VIC Australia; 11 College of Health and Human Services, Charles Darwin University, Darwin, NT Australia

## Abstract

Premature ovarian insufficiency (POI) is a common cause of infertility in women, characterised by amenorrhea and elevated FSH under the age of 40 years. In some cases, POI is syndromic in association with other features such as sensorineural hearing loss in Perrault syndrome. POI is a heterogeneous disease with over 80 causative genes known so far; however, these explain only a minority of cases. Using whole-exome sequencing (WES), we identified a *MRPL50* homozygous missense variant (c.335T > A; p.Val112Asp) shared by twin sisters presenting with POI, bilateral high-frequency sensorineural hearing loss, kidney and heart dysfunction. *MRPL50* encodes a component of the large subunit of the mitochondrial ribosome. Using quantitative proteomics and western blot analysis on patient fibroblasts, we demonstrated a loss of MRPL50 protein and an associated destabilisation of the large subunit of the mitochondrial ribosome whilst the small subunit was preserved. The mitochondrial ribosome is responsible for the translation of subunits of the mitochondrial oxidative phosphorylation machinery, and we found patient fibroblasts have a mild but significant decrease in the abundance of mitochondrial complex I. These data support a biochemical phenotype associated with *MRPL50* variants. We validated the association of *MRPL50* with the clinical phenotype by knockdown/knockout of mRpL50 in *Drosophila*, which resulted abnormal ovarian development. In conclusion, we have shown that a *MRPL50* missense variant destabilises the mitochondrial ribosome, leading to oxidative phosphorylation deficiency and syndromic POI, highlighting the importance of mitochondrial support in ovarian development and function.

**Supplementary Information:**

The online version contains supplementary material available at 10.1007/s00439-023-02563-z.

## Introduction

POI can result from premature depletion or abnormal development of the ovarian reserve. It presents clinically with absent menarche (primary amenorrhea) or ceased menstruation (secondary amenorrhea), in association with elevated FSH before the age of 40 years (Tucker et al. 2016). POI is a major cause of infertility in women, affecting up to 1 in 100 women of reproductive age (Coulam et al. 1986). This condition is highly heterogeneous and a genetic aetiology has been demonstrated for ~ 20–25% of POI patients, leaving the majority of cases unexplained (Qin et al. 2015). POI patients demonstrate a wide range of clinical phenotypes. In some cases, POI is syndromic and presents in association with other features, such as sensorineural hearing loss in Perrault syndrome, ocular muscle weakness in progressive external ophthalmoplegia (PEO), cerebellar dysfunction in ataxia telangiectasia and more (Newman et al. 2018; Tucker et al. 2016).

Several studies have demonstrated an association between mitochondrial diseases and POI. Mitochondria are the site of cellular respiration/oxidative phosphorylation (OXPHOS) and are responsible for producing the energy required for cellular functions in aerobic eukaryotic cells. Apart from supplying energy, mitochondria also have essential roles in the regulation of cellular pathways including amino acid metabolism, steroid hormone biogenesis, phospholipid biosynthesis, programmed cell death, calcium homeostasis, generation of reactive oxygen species (ROS) and antioxidant protection (McBride et al. 2006; Picard et al. 2016). The OXPHOS process not only results in the production of adenosine triphosphate (ATP) but also leads to the formation of endogenous reactive oxygen species (ROS) including oxygen radicals and hydrogen peroxide (Dunn et al. 2015). Physiologic levels of ROS are critical for healthy cell function and regulate several cellular processes (Sena and Chandel 2012). However, unregulated or mislocalised ROS can have toxic effects on cellular macromolecules, such as nucleic acids, lipids and proteins, and can be associated with mitochondrial dysfunction and related diseases (Dunn et al. 2015). Mitochondria are believed to have a key role in the female reproductive system and increased levels of ROS and impaired OXPHOS due to dysfunction of this vital organelle can result in female reproductive disorders (Lu et al. 2018; May‐Panloup et al. 2007). Along with genetic variants in mitochondrial DNA (mtDNA), decreased levels of OXPHOS activity and ATP production plus elevated levels of ROS in oocytes have also been associated with POI (Ding et al. 2019; Kumar et al. 2010). However, the role of mtDNA variants in POI pathogenesis requires further investigation of a larger population, and further studies in model organisms. Mitochondrial proteome studies in humans have shown that the vast majority of proteins localised in mitochondria (more than 1000 proteins) are encoded by the nuclear genome (Pfanner et al. 2019). Several genes involved in mitochondrial function have been implicated as causes of syndromic and/or isolated POI, highlighting that this organelle can be involved in the genetically complex aetiology of POI (Tiosano et al. 2019).

Some of the known POI-related genes in which variants impair mitochondrial function include *HARS2*, *LARS2*, *AARS2* and *RMND1* (required for the translation of mitochondrial-encoded genes) plus *POLG*, *TWNK* and *TFAM* (having roles in mtDNA replication, transcription and maintenance), *CLPP* and *CLPB* (with roles in recognition and degradation of oxidised and misfolded proteins) (Table 1) (Tiosano et al. 2019). Variants in either nuclear-encoded or mtDNA genes can lead to mitochondrial diseases. They predominantly affect organs with excessive energy demand with clinical manifestation varying from early lethal phenotypes to milder adult-onset conditions including those presenting with ovarian dysfunction (Götz et al. 2011; Taylor et al. 2014; Tucker et al. 2020). A subset of mitochondrial disorders is caused by variants in genes encoding mitochondrial ribosome (mitoribosome) proteins, RNAs and assembly factors. The mitoribosome comprises RNA components (encoded by mtDNA), as well as subunits and assembly factors (encoded by the nuclear genome). It is a 55S ribonucleoprotein complex in mammals consisting of a 39S large subunit (mt-LSU, containing a 16S rRNA, a tRNA and 52 proteins) and a 28S small subunit (mt-SSU, containing a 12S rRNA and 30 proteins) (Amunts et al. 2015; Greber et al. 2015).

**Table 1:** Mitochondrial genes associated with syndromic premature ovarian insufficiency

Gene	Protein function	Inheritance	Phenotype	References
*AARS2*	Charges mitochondrial tRNA-ala with alanine during mitochondrial translation	AR	Leukodystrophy, POI	Dallabona et al. (2014), Hamatani et al. (2016), Kiraly-Borri et al. (2019) and Lee et al. (2017)
*TWNK*	mtDNA helicase, required for mtDNA replication and maintenance	AR	Perrault syndrome, POI	Morino et al. (2014) and Yang et al. (2021)
*CLPP*	Component of a mitochondrial ATP-dependent proteolytic complex, organises mitoribosomal assembly	AR	Perrault syndrome, POI	Jenkinson et al. (2013), Szczepanowska et al. (2016) and Tucker et al. (2020)
*CLPB*	A member of the superfamily of ATP-ases associated with diverse cellular activities (AAA +), involved in protein folding regulation, DNA replication and protein degradation	AR	POI, intellectual disability, neutropenia, cataracts	Tucker et al. (2022)
*MRPS7*	A Component of the small subunit of the mitochondrial ribosome, required for the assembly of the small ribosomal subunit	AR	Perrault syndrome, POI	Kline et al. (2022)
*ERAL1*	Mitochondrial GTPase required for proper assembly of the 28S small mitochondrial ribosomal subunit	AR	Perrault syndrome, POI	Chatzispyrou et al. (2017)
*HARS2*	Charges mitochondrial tRNA-his with histidine during mitochondrial translation	AR	Perrault syndrome, POI	Karstensen et al. (2020) and Pierce et al. (2011)
*HAX1*	Required for maintaining the inner mitochondrial membrane potential	AR	Severe congenital neutropenia, POI	Carlsson et al. (2013) and Cekic et al. (2017)
*LARS2*	Charges mitochondrial tRNA-leu with leucine during mitochondrial translation	AR	Perrault syndrome, POI	Carminho-Rodrigues et al. (2020), Kosaki et al. (2018), Pierce et al. (2013) and Tucker et al. (2020)
*LRPPRC*	Required for mitochondrial mRNA stability and transport	AR	Leigh syndrome, POI	Debray et al. (2011) and Ghaddhab et al. (2017)
*POLG*	Enzyme that synthesises new mtDNA and corrects mtDNA errors	AR, AD	Progressive external ophthalmoplegia, parkinsonism, ataxia neuropathy spectrum, POI	Chen et al. (2018b) and Rahman and Copeland (2019)
*RMND1*	Required for proper functioning of mitochondria and supports translation of the mtDNA-encoded polypeptides	AR	Perrault syndrome, chronic kidney disease, POI	Oziębło et al. (2020)
*TFAM*	Mitochondrial transcription factor A, required for mtDNA maintenance and transcription	AR	Perrault syndrome, POI	De Oliveira et al. (2019), Tucker et al. (2020) and Ullah et al. (2021)
*PRORP*	A catalytic component of mitochondrial ribonuclease P, required generating functional mitochondrial tRNA molecules	AR	Perrault syndrome, POI	Hochberg et al. (2021) and Hochberg et al. (2017)

*POI* premature ovarian insufficiency, *AD* autosomal dominant, *AR* autosomal recessive

Pathological variants in several proteins of the mitoribosomal small subunit and in four proteins of the large subunit have been associated with mitochondrial diseases (summarised in Table 2) (Ferrari et al. 2021). Patients with dysfunctional mitoribosomes present with a range of phenotypes, such as Leigh syndrome (harbouring variants in *MRPS34* [MIM: 611994] and *MRPS39* [MIM: 614918]), hypertrophic cardiomyopathy (variants in *MRPS14* [MIM: 611978], *MRPS22* [MIM: 605810], *MRPL3* [MIM: 607118] and *MRPL44* [MIM: 611978]) and sensorineural hearing loss (variants in *MRPS7* [MIM: 611974],* MRPS2* [MIM: 611971] and *MRPS28* [MIM: 611990]), intellectual disability and developmental delay (variants in *MRPL24* (Di Nottia et al. 2020) and *MRPL12* [MIM: 602375]), agenesis of corpus callosum [variants in *MRPS16* (Miller et al. 2004) and *MRPS25* (Bugiardini et al. 2019)] and liver disease [variant in *MRPS23* (Kohda et al. 2016)].

**Table 2:** Nuclear genes encoding mitochondrial ribosomal proteins that are required for mitochondrial protein synthesis and associate with disease

Gene	Inheritance	Phenotype	References
*Components of mt-SSU that join in early stages of assembly*			
*MRPS2*	AR	Sensorineural hearing loss, developmental delay, and hypoglycaemia	Gardeitchik et al. (2018)
*MRPS7*	AR	Congenital sensorineural hearing loss, lactic acidemia, progressive liver and renal failure, failure of pubertal development and hypogonadism	Kline et al. (2022) and Menezes et al. (2015)
*MRPS22*	AR	Cardiomyopathy, muscle hypotonia, fatal lactic acidosis, dysmorphism, Leigh syndrome-like lesions, brain abnormalities, POI	Baertling et al. (2015), Chen et al. (2018a), Kılıç et al. (2017) and Smits et al. (2011a, b)
*MRPS23*	AR	Liver disease	Kohda et al. (2016)
*MRPS28*	AR	Sensorineural hearing loss, sever developmental delay and dysmorphism	Pulman et al. (2019)
*MRPS34*	AR	Neurodevelopmental defects, Leigh syndrome	Lake et al. (2017a, b)
*PTCD3 (MRPS39)*	AR	Leigh syndrome	Borna et al. (2019)
*Component of mt-SSU that join in late stages of assembly*			
*MRPS14*	AR	Hypertrophic cardiomyopathy, infantile mental retardation, hyperlactatemia, cachexia	Jackson et al. (2019a, b)
*MRPS25*	AR	Partial agenesis of corpus callosum, short stature, muscle weakness, dysphagia	Bugiardini et al. (2019)
*Component of mt-SSU providing the basis for whole mt-SSU assembly*			
*MRPS16*	AR	Fatal neonatal lactic acidosis, agenesis of corpus callosum, dysmorphism	Miller et al. (2004)
*Components of mt-LSU that join in early stages of assembly*			
*MRPL3*	AR	Hypertrophic cardiomyopathy, growth failure, liver dysfunction, hepatomegaly	Galmiche et al. (2011a, b)
*MRPL12*	AR	Delayed growth, neurologic deterioration	Serre et al. (2013)
*MRPL44*	AR	Hypertrophic cardiomyopathy, liver steatosis	Carroll et al. (2013) and Distelmaier et al. (2015a, b)
*Component of mt-LSU that joins during the intermediate/late phase of assembly*			
*MRPL24*	AR	Cerebellar atrophy, choreoathetosis of limbs and face, intellectual disability	Di Nottia et al. (2020)

*Mt-SSU* mitochondrial small subunit, *mt-LSU* mitochondrial large subunit, *AR* autosomal recessive

Homozygous variants in *MRPS22* (Chen et al. 2018a) have been reported in non-syndromic POI patients, whereas variants in *MRPS7* (Menezes et al. 2015) have been described in a patient with failure of pubertal development and hypogonadism, emphasizing the role of these genes in ovarian development. We have recently identified two heterozygous variants in *MRPS7* in a POI patient with small ovaries presenting with secondary amenorrhea and sensorineural hearing loss which are indicative of Perrault syndrome (Kline et al. 2022).

In the current study, we report the first pathological variant in *MRPL50* (mitochondrial ribosomal protein L50), encoding a component of the mitochondrial ribosomal large subunit. The homozygous missense variant was identified in dizygotic twin sisters presenting with amenorrhea, sensorineural hearing loss, chronic kidney disease and left ventricular hypertrophy. A range of functional studies were undertaken to validate the variant pathogenicity including analysis of patient fibroblasts and *Drosophila* modelling. The *MRPL50* variant is likely causative of syndromic POI, highlighting the crucial role of mitochondria in the development and function of ovaries.

## Materials and methods

### Patient clinical information

The affected patients were dizygotic twins of Indian descent from a third-degree consanguineous marriage. The first-born twin (Twin 1) presented at 26 years of age with secondary amenorrhea and history of hearing loss. She attained menarche at 17 years of age and had two cycles after which she was amenorrhoeic. She had some breast development and noticed no recent regression in breast size. She had noticed difficulty in hearing for the past 2 years. There was no history of developmental delay or cognitive deficit. On examination, she had normal stature (ht-157 cm [25–50th centile]) with no dysmorphic features. She was Tanner Stage 5 for breast development, stage 1 for pubic hair and zero for axillary hair (B_5_P_1_A_0_). She is hypertensive with a blood pressure of 150/100 mm Hg. She has long tapering fingers and toes. Investigation showed normal karyotype (46,XX) with evidence of hypergonadotropic hypogonadism (FSH-155 IU/L, LH-44 IU/L). Pure tone audiometry showed bilateral normal-profound steeply sloping sensorineural hearing loss with normal hearing at the lowest frequencies tested and profound hearing loss at the highest frequencies tested (Supplementary Fig. S1). Abdominal ultrasound showed non-visualization of ovaries, a hypoplastic uterus and bilaterally contracted kidneys. Echocardiography showed concentric left ventricular hypertrophy with normal left ventricular function. Urine microscopy showed trace proteinuria with 24-h quantification of 150 mg (Table 3).

**Table 3:** Summary of patients’ clinical information

Patient	Karyotype	Reproductive phenotype					Hearing	Kidney	Heart	Other
		Age of diagnosis	Menstrual status	Hormones	Imaging	Puberty staging				
Twin 1	46,XX	26	Secondary amenorrhea (3 cycles only)	FSH: 155 IU/L, LH: 44 IU/L	Ovaries not visualised, hypoplastic uterus	B_5_P_1_A_0_	Bilateral normal-profound steeply sloping sensorineural hearing loss, onset age 24	Bilaterally contracted kidneys	Left ventricular hypertrophy	Long tapering fingers and toes
								Hypertension (150/100)		
								Proteinuria		
Twin 2	46,XX	26	Primary amenorrhea	FSH: 102 IU/L, LH: 47.5 IU/L	Ovaries and uterus not visualised	B_3_P_1_A_0_	Bilateral normal-profound steeply sloping sensorineural hearing loss, onset age 24	Bilaterally contracted kidneys	Left ventricular hypertrophy	Long tapering fingers and toes
								Hypertension (160/100)		
								Proteinuria		

The second-born twin (Twin 2) presented at 26 years of age with primary amenorrhea and also had a history of hearing loss. She had normal breast development. Like Twin 1, hearing difficulties were noted at 24 years of age and there were no signs of intellectual disability. On examination, she had normal stature (ht-152.5 cm [10–25th centile]) with no dysmorphic features and she was Tanner stage 3 for breast development, 1 for pubic hair and zero for axillary hair (B_3_P_1_A_0_). She is also hypertensive with blood pressure of 160/100 mm Hg and has the same distinctive long tapering fingers and toes as Twin 1. Investigation showed normal karyotype (46,XX) and evidence of hypergonadotropic hypogonadism (FSH-102 IU/L, LH-47.5 IU/L). Pure tone audiometry found bilateral normal-profound steeply sloping sensorineural hearing loss. Abdominal ultrasound showed non-visualization of uterus and ovaries, and bilaterally contracted kidneys. Echocardiography showed concentric left ventricular hypertrophy with normal left ventricular function. Urine microscopy showed trace proteinuria with 24-h quantification of 493 mg (Table 3).

### Whole-exome sequencing

Whole-Exome Sequencing (WES) of DNA from Twin 1 was performed at the Victorian Clinical Genetics Services (VCGS). Exome capture with Agilent SureSelect Human All Exon V6 and sequencing on an Illumina NovaSeq 6000 were performed. WES data were processed by Cpipe pipeline (Sadedin et al. 2015) and analysed using SeqR (https://seqr.broadinstitute.org/). We used two different approaches to analyse WES data as reported previously (Tucker et al. 2019), the first was gene-centric and focussed on gene priority using POI candidate genes [adapted from Tucker et al. (Tucker et al. 2019)] and the second was variant-centric and focussed on variant priority. For gene-centric analysis, we considered moderate–high priority and high-quality [minor allele frequency (MAF) < 0.001)] variants in our previously established candidate POI gene list, as well as the Kidneyome SuperPanel and the Deafness (Isolated and Complex) gene list from PanelApp-AUS (https://panelapp.agha.umccr.org/) (Supplementary File S1). For variant-centric analysis, moderate–high priority (MAF < 0.001) bi-allelic variants in any gene or high priority loss-of-function variants (MAF < 0.0001) were considered. We also performed copy number variant (CNV) analysis on WES data as previously described (Sreenivasan et al. 2022).

To predict the variant pathogenicity in silico, we used several online tools including Polyphen2 (https://genetics.bwh.harvard.edu/pph2), SIFT/Provean (https://provean.jcvi.org/), Mutation Taster (https://www.mutationtaster.org/) and CADD (Combined Annotation-Dependent Depletion) score (https://cadd.gs.washington.edu/snv). GnomAD (https://gnomad.broadinstitute.org/, accessed in November 2022) was used for analysing the minor allele frequency (MAF) as well as the tolerance of genes to missense and/or loss-of-function variation. Multiz Alignments of 100 vertebrates (UCSC Genome Browser https://genome.ucsc.edu/) were used to analyse the conservation of affected residues.

### Sanger sequencing

For variant validation and familial segregation studies, exon 2 of *MRPL50* was amplified via PCR using the following primers: Forward: 5′ GGCAAGATTTGGTCTGGTGT 3′ and Reverse: 5′ TGCTCAGGGAAAGACAGTGA 3′. Sanger sequencing was performed using BigDye v3.1 Terminators (Applied Biosystems) and ABI 3130X.

### Human cell culture

Skin biopsies from Twin 2 were obtained and a fibroblast cell line was generated. Fibroblasts from two different healthy controls (HC1 and HC2) and one disease control (DC) (harbouring likely pathogenic variants in *MRPL39*, ClinVar Accession IDs VCV001676672.1 and VCV001676674.2) were also studied. Peripheral blood mononuclear cells were used to generate patient lymphoblast lines.

Cells were grown at 37 ℃ and 5% CO_2_ in Dulbecco’s modified Eagle’s medium (DMEM; Thermo Fisher Scientific) supplemented with 10% (v/v) foetal bovine serum (FBS, Thermo Fisher Scientific) and 1% (v/v) penicillin–streptomycin (Thermo Fisher Scientific) (fibroblasts) or RPMI 1640 media with 20% FBS (lymphoblasts) using aseptic standard cell culture procedures.

### Expression analysis

RNA was extracted using the ReliaPrep RNA Cell Miniprep System (Promega). cDNA was synthesized using the GoScript reverse transcriptase system (Promega). qRT-PCR was performed with GoTaq qPCR Master Mix (Promega) on the LightCycler480 (Roche) with GAPDH as the reference gene. The efficiency (E) of MRPL50 and GAPDH was established using a cDNA dilution series. The relative expression of MRPL50 to GAPDH was determined [E(GAPDH) ^Ct(GAPDH)^/E(MRPL50)^Ct(MRPL50)^] and was normalized to the average of four controls. Three independent qRT-PCR experiments were performed with samples in triplicate. Primer sequences are available upon request.

### Western blotting

Proteins were extracted from cultured primary fibroblast cells with extraction buffer A (MitoSciences) as previously described (Calvo et al. 2010). Protein lysates were quantified using the Pierce BCA protein assay kit (ThermoScientific, 23227), accompanied by BSA protein standards.

Five micrograms of total protein lysate was prepared with 2X Solubilisation buffer (125 mM Tris (pH 8.0), 40% glycerol, 4% SDS, 100 mM DTT, bromophenol blue) and Complete mini protease inhibitor (Roche). Samples were heated at 94 °C for 3 min then run on 10% Bis–Tris NuPAGE gels (ThermoFisher) with MOPS or MES buffer (ThermoFisher). Proteins were transferred to PVDF membranes, blocked using 5% skim milk powder/TBST and incubated with primary antibodies including two different anti-MRPL50 polyclonal antibodies (1:500, Invitrogen, PA5-39246) and (1:500, Invitrogen, PA5-101675), anti-MRPL13 polyclonal antibody (1:500, Invitrogen, PA5-96487), anti-MRPL44 polyclonal antibody (1:1000, Proteintech, 16394-1-AP), anti-MRPL39 polyclonal antibody (1:10,000, Proteintech, 28165-1-AP), anti-MRPL9 polyclonal antibody (1:2400, Proteintech, 15342-1-AP), anti-MRPS17 polyclonal antibody (1:1000, Proteintech, 18881-1-AP), anti-MRPS7 polyclonal antibody (1:10,000, Proteintech, 26828-1-AP) and Total OXPHOS Human WB antibody Cocktail (1:500, Abcam, ab110411) overnight at 4 °C. Blots were washed with TBST and incubated with secondary antibodies, swine anti-rabbit HRP (1:20,000 DAKO P0399) and donkey anti-mouse from (1:20,000 DAKO P0260) at room temperature for 2 h. Anti-alpha Tubulin (HRP) (1:5000, Abcam, ab40742) was used as a loading control.

Proteins were detected using the Amersham ECL Prime Western blotting detection system (RPN2132) and visualised with the ImageQuant software (GE Healthcare Life Sciences ImageQuant LAS 4000).

### Quantitative proteomics

A total of 50 µg (Pierce BCA Assay Kit; Thermo Fisher Scientific) fibroblast cells (*MRPL50* Twin 2, Control 1, Control 2 in triplicate; *MRPL39* DC in triplicate and Control 3, Control 4 and Control 5 in singlicate) were solubilised in 1% [w/v] sodium deoxycholate, 100 mM Tris pH 8.1, 40 mM 2-chloroacetamide, and 10 mM Tris(2-carboxyethyl)phosphine hydrochloride (TCEP; BondBreaker; Thermo Fisher Scientific) for 5 min at 99 °C with 1500 rpm shaking followed by 15 min sonication in a water bath sonicator. Proteins were digested with trypsin (Thermo Fisher Scientific) at a 1:50 trypsin to protein ratio at 37 °C overnight. The supernatant was transferred to stagetips containing 3 × 14 G plugs of 3 M Empore SDB-RPS substrate (Sigma) as described previously (Kulak et al. 2014; Stroud et al. 2016). Isopropanol 99% (v/v) and 1% trifluoroacetic acid (TFA) (v/v) was added to the tip before centrifugation at 3000 × *g* at room temperature. Stagetips were washed first with isopropanol (99% [v/v]) and TFA (1% [v/v]) solution and then subjected to a second wash containing 0.2% (v/v) TFA. Peptides were eluted in 80% (v/v) acetonitrile and 1% (w/v) NH_4_OH and acidified to a final concentration of 1% (v/v) TFA before drying in a CentriVap Benchtop Vacuum Concentrator (Labconco). Peptides were reconstituted in 0.1% TFA and 2% acetonitrile (ACN) for analysis by liquid chromatography (LC)–MS/MS. LC–MS/MS was carried out on an Orbitrap Eclipse mass spectrometer (Thermo Fisher Scientific) with a nanoESI interface in conjunction with an Ultimate 3000 RSLC nanoHPLC (Dionex Ultimate 3000). The LC system was equipped with an Acclaim Prepmap nano-trap column (Dionex C18; 100 Å, 75 μM × 50 cm). The tryptic peptides were injected into the enrichment column at an isocratic flow of 6 μl/min for 5 min applied before the enrichment column was switched in-line with the analytical column. The eluents were 5% dimethyl sulfoxide (DMSO) in 0.1% (v/v) formic acid (solvent A) and 5% DMSO in 100% (v/v) CH_3_CN and 0.1% (v/v) formic acid (solvent B). The flow gradient was (1) 0–6 min at 3% B, (2) 6–95 min at 3–23% B, (3) 95–105 min at 23–40% B, (4) 105–110 min at 40–80% B, (5) 110–115 min at 80% B, (6) 115–115.1 min at 80–3% B, (7) 115.1–125 min at 3–0% B. Equilibration was performed with 3% B for 10 min before the next sample injection. The mass spectrometer was operated in the data-dependent mode with a targeted inclusion list containing predicted peptides from the 13 mitochondrial DNA-encoded proteins as described previously (Hock et al. 2020). Full MS1 spectra were acquired in positive mode, at 120,000 resolution (Orbitrap), AGC target of 4e^5^, and maximum IT time of 50 ms. A loop count of 15 on the most intense targeted peptides was used to isolate precursors for MS/MS. The isolation window was set to 1.6 m/z and precursors fragmented using a collision energy of 30. MS2 resolution was at 15,000, AGC target at 5e^5^, and maximum IT time of 100 ms. Dynamic exclusion was set to be 30 s.

Raw files were processed using the MaxQuant platform (version 1.6.10.3) (Cox and Mann 2008) and searched against the UniProt human database containing canonical and isoforms (November 2019) using default settings for a label-free quantitation (LFQ) experiment with match between runs enabled. The proteinGroups.txt output from the search was processed in Perseus (version 1.6.14.0) (Tyanova et al. 2016). Briefly, entries “Only identified by site,” “Reverse,” and “Potential contaminant” were removed from the data set. Log_2_-transformed LFQ intensities were grouped (controls, patient) and filtered to have at least two valid values in both groups. Mitochondrial proteins were annotated using MitoCarta 3.0 database (Rath et al. 2021) through matching by protein IDs. A two-sample *t*-test was performed between groups using *p* value for truncation (threshold *p *value < 0.05) using MitoCarta-positive entries. Volcano plots were generated via scatter plot by selecting “Student’s *t*-test difference” as *x*-axis and “-Log Student’s *t*-test *p *value” as *y*-axis. Topographical mapping of log_2_ fold-changes from *t*-test was performed as described previously (Stroud et al. 2016) onto the mitoribosome structure (PDB id: 3J9M). Relative Complex Abundance (RCA) plots were generated as described previously (Lake et al. 2017a) with some modifications. Briefly, raw LFQ intensity values for each OXPHOS and mitoribosome subunit were imported into Prism 8 software. Mean values for each subunit in each complex were obtained and a ratio paired *t*-test was performed to determine the significance of the overall complex abundance. The ratios were log_10_-transformed for plotting.

### *Drosophila* mRpL50 knockdown/knock-out models

#### Fly stocks and husbandry

UAS-GAL4; GAL4::VP16-nos.UTR, generated (from BL4937 and BL5938) driver and referred to as Nos-Gal4, *w*^*1118*^ control strain (BL5905) and Cas9/CyO; Lpp-Gal4/TM6B (3rd chromosome insert, BL67078) *Drosophila* stocks were obtained from the Bloomington *Drosophila* Stock Center. Tj-GAL4 (DGRC104055) was obtained from the Kyoto Stock Centre. RNAi fly strains mRpL50RNAi (V15199) (CG9236, 3rd chromosome insert) and mRpL50RNAi (V106402) (CG8612, 2nd chromosome insert) were obtained from the Vienna *Drosophila* Resource Centre. mRpL50-gRNA lines were generated by BestGene Inc. Co. All fly stocks were maintained on standard molasses-based food at 25 °C. Two to three days after mating to GAL4 drivers, larvae were transferred to 29 °C for further analysis following eclosion.

### Generation of mRpL50-RNAi *Drosophila* knockdown

To generate *mRpL50* knockdown (KD) flies, two RNAi fly strains, *mRpL50*^RNAi^ (V15199) and *mRpL50*^RNAi^ (V106402) were obtained. An *mRpL50*^double−RNAi^ (V15199/V106402) knockdown strain harbouring both RNAi transgenes was also generated to increase the KD efficiency of *mRpL50*. Crosses were performed with both single and double RNAi strains separately with the somatic cell-specific (Traffic jam-Gal4) and germline-specific (Nanos-Gal4) Gal4 drivers to produce the offspring harbouring tissue-specific KD of *mRpL50* expression (Crosses summarised in Table 4). Ovaries and testes of offspring were dissected and immunostained using germ line-specific and somatic cell-specific markers.

**Table 4:** Different crosses amongst the somatic cell and germline-specific Gal4 drivers with mRpL50-RNAis and UAS Cas9-*mRpL50*-gRNA

Gal4 drivers	*mRpL50*-specific drivers	Gonadal structure in *mRpL50*-KD/KO offspring
Traffic jam-Gal4 (Somatic cell-specific)	*mRpL50*-Single RNAi (V15199)	No obvious phenotype
	*mRpL50-*Single RNAi (V106402)	*mRpL50-*somatic-KD flies with small and undeveloped gonads (failure to produce organised ovarian egg chambers, over-proliferation of early germ cells in testes)
	*mRpL50-*Double RNAi (V15199/V106402)	*mRpL50-*somatic-KD flies with small and undeveloped gonads (failure to produce organised ovarian egg chambers, over-proliferation of early germ cells in testes)
	UAS Cas9-*mRpL50*-specific gRNA	*mRpL50-*somatic-KO flies with small and abnormal gonadal structure and development (failure to produce organised ovarian egg chambers, over-proliferation of early germ cells in testes)
Nanos-Gal4 (Germline-specific)	*mRpL50*-Single RNAi (V15199)	No obvious phenotype
	*mRpL50-*Single RNAi (V106402)	No obvious phenotype
	*mRpL50-*Double RNAi (V15199/V106402)	No obvious phenotype
	UAS Cas9-*mRpL50*-specific gRNA	*mRpL50-*germline-KO flies with small and undeveloped gonads in *mRpL50-*gemline-KO flies, no germ cells were present in both gonads

### Generation of CRISPR/Cas9-mediated mRpL50-gRNA knockout strain

To establish the tissue-specific mRpL50 knock-out (KO) *Drosophila* strain, an MRPL50-specific double gRNA construct was generated by cloning tandem gRNA expression constructs into pCFD4 plasmid. The CRISPR Optimal Target Finder online tool (targetfinder.flycrispr.neuro.brown.edu) was used to design gRNA sequences that target two flanking sites of a predicted pathogenic *MRPL50* variant. We confirmed that there were no identical sites in the fly genome, as well as no polymorphism in the targeted regions in different *Drosophila* strains. These gRNA sequences were incorporated into forward and reverse primers that were then used for amplification with a high-fidelity polymerase and pCFD4 plasmid as a template. The pCFD4 vector was digested with BbsI restriction enzyme. The desired DNA fragments were gel-purified (PCR 600 bp; Backbone 6.4 kb). Using the Gibson Assembly method, the PCR product and vector backbone were assembled and transformed into the XL-1 Blue competent bacteria. Ultimately, Sanger sequencing was used to verify the inserted sequences using the following sequencing primer: GACACAGCGCGTACGTCCTTCG. Plasmid DNA from sequence-verified colonies was isolated using the NucleoSpin Plasmid Transfection-grade kit. Using the mRpL50-double gRNA construct, the transgenic *Drosophila* strains expressing mRpL50-gRNA ubiquitously were generated by BestGene Inc. Co.

The ubiq-mRpL50-gRNA *Drosophila* transgenic line was crossed to a tissue-specific UAS-Cas9 strain to produce a UAS-Cas9, *mRpL50*-gRNA strain. Then, this strain was mated with TJ-Gal4 and Nanos-Gal4 drivers separately to produce somatic cell-specific and germline-specific Gal4, UAS-Cas9, *mRpL50*-gRNA KO offspring resulting in CRISPR mutagenesis in the organ of our interest (Table 4).

### Immunostaining and imaging

The ovaries and testes were dissected from 5- to 6-day-old flies and fixed in 4% formaldehyde diluted in PBT [PBS + 0.2% Triton X-100 (Sigma)] for 15 min. The gonads were washed in PBT (3 × for 10 min) and blocked in PBTH (5% Normal Horse Serum diluted with PBT) for one hour. The blocked gonads were then incubated overnight at 4 °C in primary antibodies including goat anti-Vasa (dc-13) (1:100, Santa Cruz Biotechnology), mouse anti-FasIII [1:50, Developmental Studies Hybridoma Bank (DSHB)] and chicken anti-GFP (1:2000, Abcam). On the following day, samples were washed and incubated at room temperature for 2 h in secondary antibodies (Thermo Fisher Scientific) including Donkey anti-Mouse Alexa Fluor 647, Donkey anti-goat Alexa Fluor 594, Donkey anti-chicken Alexa Fluor 488. All secondary antibodies were used at a dilution of 1:500. Samples were washed further in PBT and mounted on slides in Prolong™ Gold Antifade Reagent with DAPI (Invitrogen). Imaging was performed on a Zeiss LSM800 confocal microscope and optimised to acquire overlapping sections using serial optical sections (*z*-stacks). Images were processed using FIJI/ImageJ software.

## Results

### Identification of a homozygous missense variant in *MRPL50*

**Fig. 1:**
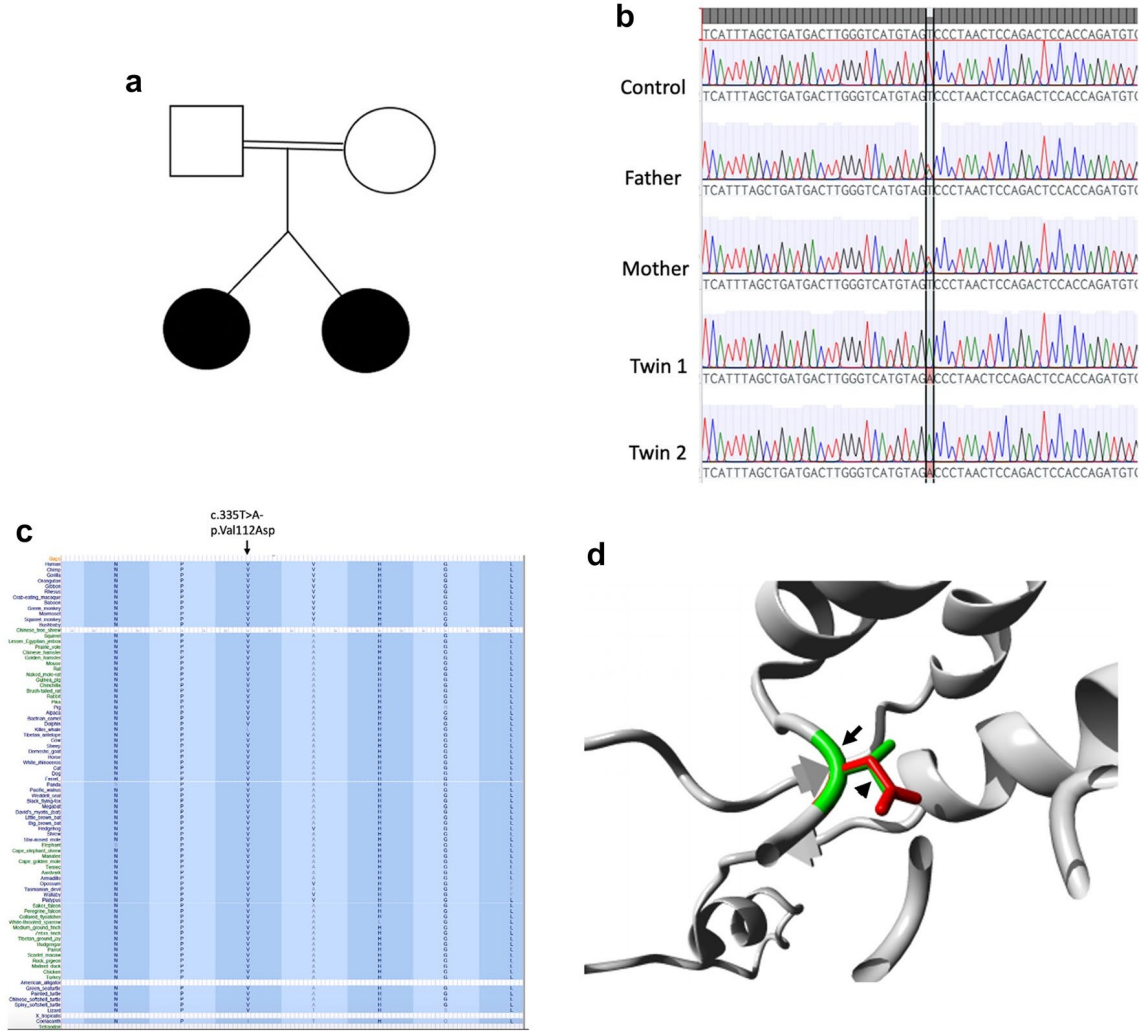
Pathogenic variants in *MRPL50*. **a** Familial pedigree of patients. Black circles represent affected dizygotic twins. **b** Sanger sequencing demonstrates segregation of the *MRPL50* variant with disease. **c** Multiz alignment indicating conservation of the affected residue. **d** The 3D structure of MRPL50 and the effect of missense variant were visualized using HOPE database. Substitution of Val112 (shown in green with an arrow) with Asp residue (shown in red with an arrowhead). The variant introduces a less hydrophobic residue which is predicted to affect both multimer contacts and multimerization. Subsequently, the variant is likely to lead to the loss of hydrophobic interactions with other molecules present on the surface of the protein

WES studies based on gene-centric and variant-centric analysis detected 22 variants of interest in the proband (Tables 5, 6). These variants were further considered for their likely role in causing the patient phenotype. There were five variants in POI candidate genes detected by gene-centric analysis. These variants were discounted because two (*AARS2* and *WRN*) were single heterozygous variants in genes associated with autosomal recessive syndromic POI and the remaining genes/variants (*HYAL1*, *CBX3* and *VAX1*) had only weak association with potential POI pathogenicity. Two heterozygous variants of interest were identified in deafness-related genes, one of which was discounted because it was inherited from an unaffected father (*MAP1B*) and the other of which was discounted because the patient phenotype did not match that associated with the gene (thiamine responsive megaloblastic anaemia syndrome, *SLC19A2*) (Supplementary Table S1). Eight heterozygous variants were identified in nephropathy-associated genes (*CLCN2, C5orf42, GSN, LMX1B, CEP164, PKD1, ITGB4, and KANK2*), but each could be discounted having been inherited from an unaffected parent (Supplementary Table S2). Regarding the variant-centric analysis, six genes had recessive-type variants and eleven genes had high priority predicted loss-of-function (LOF) variants. Variants were discounted in many genes (*GDP1, ADAM8, DPYSL4, LIPA, SLC35D2, SLC44A4, ARHGEF37, and GK2*) because they were heterozygous in both the affected patient and the unaffected mother. The other variants were not considered likely causative due to lack of genotype/phenotype correlation and/or no evidence of function of the genes in ovarian development. We considered a homozygous *MRPL50* variant [Chr9:101390608A > T, NM_019051.3:c.335T > A; p.(Val112Asp)] the top candidate given previous reports describing *MRPS7* (Kline et al. 2022; Menezes et al. 2015) and *MRPS22* (Chen et al. 2018a) in association with POI. Sanger sequencing confirmed segregation of the *MRPL50* variant with disease with both affected dizygotic twin siblings being homozygous and both unaffected parents being heterozygous for the variant as showing in their pedigree (Fig. 1a, b). This variant is absent in population databases (gnomAD, ExAC) and predicted pathogenic by online algorithms including SIFT (score 2.97), PROVEAN (score − 6.52), Polyphen (Score 1.0) and MutationTaster (score 0.1). The variant affects an evolutionarily conserved residue (UCSC Genome Browser) suggesting that changes at this site are likely to be detrimental to protein function (Fig. 1c). Given the 3D structure of MRPL50 is known, the effects of the missense variant were modelled using HOPE (http://www.cmbi.ru.nl/hope/) (Venselaar et al. 2010). The variant residue differs in size, charge and hydrophobicity compared to the wildtype residue. The wildtype residue forms a hydrogen bond and salt bridge with serine at position 68, both interactions of which would be disrupted by the variant. The variant residue is also involved in multimer contact and multimerization is likely altered by the variant. These changes are likely to influence the stability of the MRPL50 protein (Fig. 1d).

**Table 5:** Genes identified with variants of interest after filtration

Gene-centric analysis	Variant-centric analysis (recessive)	Variant-centric analysis (LOF)
Moderate-high priority, MAF < 0.001, high-quality, POI candidate genes (Supplementary File 1, Table S1)	Moderate-high priority, MAF < 0.001, high-quality, recessive inheritance (Supplementary File 1, Table S2)	high priority, MAF < 0.0001, high-quality (Supplementary File 1, Table S3)
*HYAL1*, *AARS2*, *CBX3*, *WRN*, *VAX1*	*MRPL50*, *OR4P4*, *GANC*, *PIEZO2*, *MPPE1*, *MICAL3*	*AKR7A3*, *TTC31*, *GK2*, *ARHGEF37*, *SLC44A4*, *SLC35D2*, *LIPA*, *DPYSL4*, *ADAM8*, *GPD1*, *LINC01483*

**Table 6:** Summary of variants identified after filtering of WES data in the proband

Gene	Function	Variant	gDNA (hg38)	cDNA	Protein	Polyphen (cutoff − 2.5)	SIFT (cutoff 0.05)	Mutation Taster	FATHMM	CADD (22ut—of > 1.75)	Disease associated with the gene
*MRPL50*	Component of the mitochondrial ribosomal large subunit, involved in translating mitochondrial mRNAs	Homozygous missense	Chr9:101390608A > T	c.335T > A	p.(Val112Asp)	Damaging (Score 1.0)	Damaging (score 2.97)	Damaging (score 0.1)	Tolerated	29.1	
*OR4P4*	Odorant receptor	Missense	Chr11:55639024A > T	c.667A > T	p.(Ile223Phe)		Damaging		Tolerated	14.37	
*GANC*	Has alpha-glucosidase activity	Missense	Chr15:42310356G > T	c.796G > T	p.(Gly266Cys)	Damaging	Damaging	Damaging	Damaging	26.4	
*PIEZO2*	Component of a mechanosensitive cation channel	Missense	Chr18:10807261T > C	c.931A > G	p.(Lys311Glu)		Tolerated		Tolerated	23.6	Marden–Walker syndrome and arthrogryposis, distal, type 5 (autosomal dominant, Heterozygous missense mutations, MIM: 613629)
*MPPE1*	Metallophosphoesterase required for transport of GPI-anchor proteins from the endoplasmic reticulum to the Golgi	Missense	Chr18:11897072T > C	c.193A > G	p.(Thr65Ala)				Tolerated	8.096	Multiple Congenital Anomalies-Hypotonia-Seizures Syndrome, Multiple Congenital Anomalies-Hypotonia-Seizures Syndrome 2
*MICAL3*	Monooxygenase that promotes depolymerization of F-actin	Missense	Chr22:17886041G > A	c.2078C > T	p.(Pro693Leu)		Tolerated	Damaging	Tolerated	26.5	Bardet–Biedl Syndrome 17
*AKR7A3*	Aldo–keto reductase which involved in the detoxification of aldehydes and ketones	Frameshift	Chr1:19286345AGTGGAATG > A	c.234_241delCATTCCAC	p.(Ile79ValfsTer8)					29.5	Cytochrome P450 Oxidoreductase Deficiency
*TTC31*	A part of Tetratricopeptide-like helical domain superfamily, mediates protein—the assembly of multiprotein complexes	Splice_acceptor	Chr2:74492003G > T	c.877-1G > T	p.?			Damaging		22.4	
*GK2*	Has glycerol kinase activity which is involved in several metabolic processes	Stop_gained	Chr4:79407708G > A	c.493C > T	p.(Gln165Ter)			Damaging		35	Glycerol Kinase Deficiency and Hypoadrenocorticism, Familial
*ARHGEF37*	A guanine nucleotide exchange factor, involved in regulation of catalytic activity	Stop_gained	Chr5:149616646C > T	c.538C > T	p.(Gln180Ter)					37	Epidermolysis Bullosa Simplex 1a, Generalized Severe (EBS1A)
*SLC44A4*	A transmembrane transport protein that is sodium-dependent, involved in the uptake of choline by cholinergic neurons	Stop_gained	Chr6:31874814C > T	c.375G > A	p.(Trp125Ter)					35	Deafness, Autosomal Dominant 72 (Heterozygous missense mutation, MIM: 606107) and Autosomal Dominant Non-Syndromic Sensorineural Deafness Type Dfna
*SLC35D2*	Encodes nucleotide sugars, donor substrates for glycosyltransferases located in the lumen of the Golgi and endoplasmic reticulum	Frameshift	Chr9:96383524GC > G	c.110delG	p.(Cys37SerfsTer13)						Dicarboxylic Aminoaciduria and Thiamine-Responsive Megaloblastic Anaemia Syndrome
*LIPA*	Encodes a lysosomal acid lipase (lipase A)	Splice_acceptor	Chr10:89223832T > A	c.676-2A > T	p.?			Damaging		26.4	Lysosomal Acid Lipase Deficiency and Cholesterol Ester Storage Disease (Autosomal recessive, Homozygous mutations, MIM: 613497)
*DPYSL4*	Involved in nervous system development through filamin binding activity	Splice_donor	Chr10:132192842TG > T	c.313 + 1delG	p.?						Alternating Esotropia
*ADAM8*	Encodes a member of a disintegrin and metalloprotease domain (ADAM) family, Possible involvement in extravasation of leukocytes	Splice_donor	Chr10:133274795C > A	c.345 + 1G > T	p.?					2.436	Asthma and Breast Large Cell Neuroendocrine Carcinoma
*GPD1*	Has glycerol-3 phosphate dehydrogenase activity, play a role in carbohydrate and lipid metabolism	Splice_acceptor	Chr12:50106285CAG > C	c.361-2_361-1delAG	p.?					26.4	Transient infantile hypertriglyceridemia (Autosomal recessive, Homozygous splice site mutation, MIM: 138420)
*LINC01483*	Long intergenic non-protein coding RNA 1483	Splice_donor	Chr17:69624753G > A	n.239 + 1G > A	p.?					14.03	
*HYAL1*	A lysosomal hyaluronidase, May be involved in promoting tumour progression	Missense	Chr3:50300677G > A	c.1114C > T	p.(Arg372Cys)		Damaging		Tolerated	25.4	Mucopolysaccharidosis, Type IX (Autosomal recessive, Compound heterozygote mutations, MIM: 607071) and Natowicz Syndrome
*AARS2*	Aminoacyl-tRNA synthetases which charges mitochondrial tRNA-ala with alanine during mitochondrial translation	Inframe_deletion	Chr6:44302127AGCTCCC > A	c.2525_2530delGGGAGC	p.(Arg842_Glu843del)						Combined Oxidative Phosphorylation Deficiency 8, Progressive Leukoencephalopathy with Ovarian Failure (Autosomal recessive, Homozygous and compound heterozygote mutation, MIM: 612035)
*CBX3*	Involved in transcriptional silencing in heterochromatin-like complexes	Splice_donor	Chr7:26208556G > GC	c.330 + 1_330 + 2insC	p.?					25.9	Hutchinson-Gilford Progeria Syndrome and Hyperoxaluria, Primary, Type I
*WRN*	Has ATP-dependent DNA-helicase activity, Involves in DNA repair, replication, transcription and telomere maintenance	Missense	Chr8:31173043G > C	c.4240G > C	p.(Gly1414Arg)		Tolerated		Tolerated	12.58	Werner syndrome (Autosomal recessive, splice junction, homozygous and nonsense mutations, MIM: 277700) and Bloom Syndrome
*VAX1*	A transcription factor that may function in dorsoventral specification of the forebrain	Missense	Chr10:117134102T > C	c.911A > G	p.(Gln304Arg)	Damaging	Tolerated	Damaging	Damaging	26.9	Microphthalmia, Syndromic 11 (Autosomal recessive, Homozygous missense mutation, MIM: 604294) and Colobomatous Microphthalmia

### Mitoribosomal protein expression in a patient fibroblast cell line

**Fig. 2:**
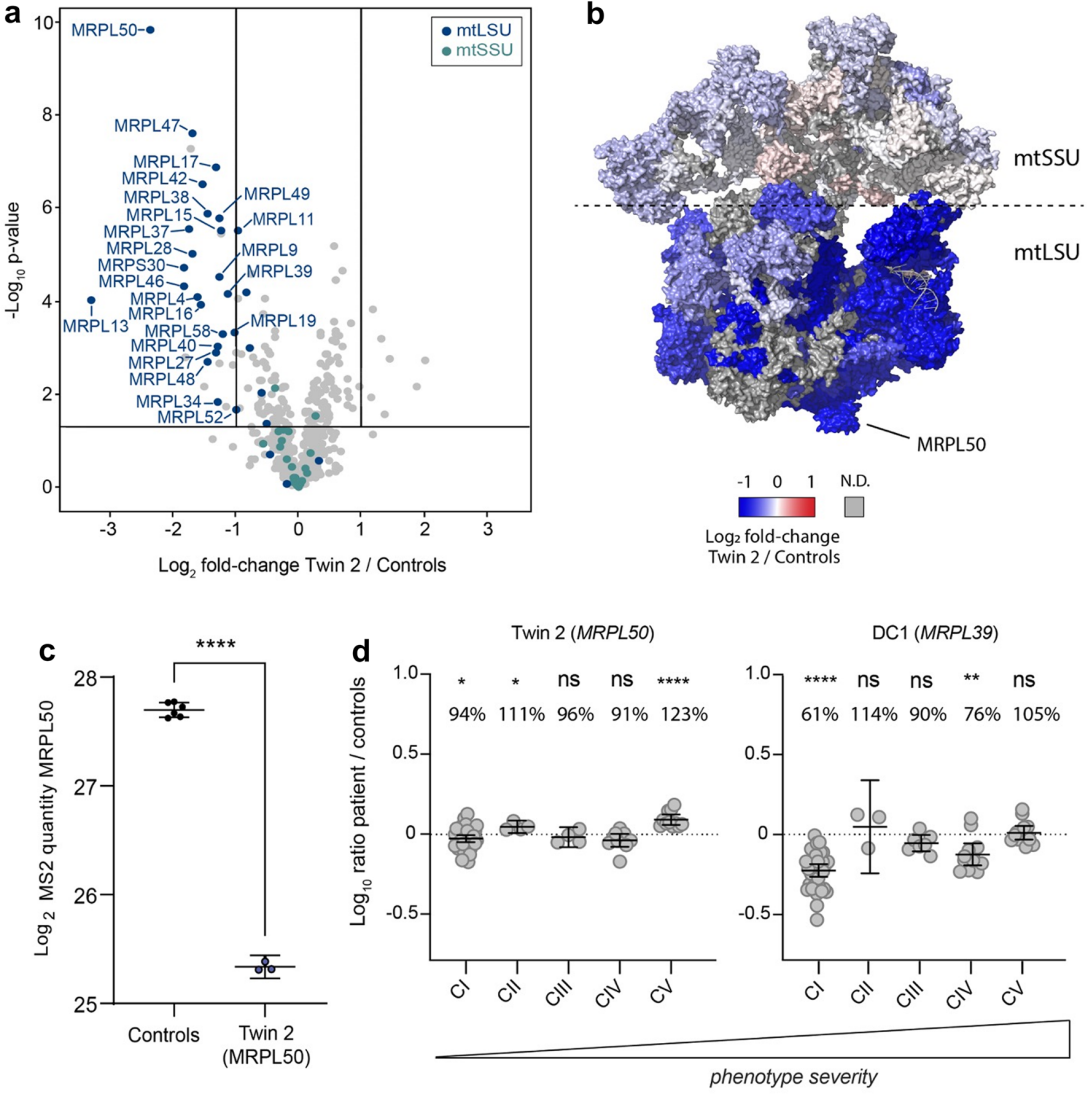
Quantitative proteomic analysis demonstrates disrupted proteins of large subunit of the mitochondrial ribosome **a** volcano plot depicting the difference in the relative abundance of mitochondrial proteins in the *MRPL50* Twin 2 patient compared to controls (*N* = 2) after *t*-test analysis. Each dot represents a protein that is marked with its respective gene name. Significance bars were set to twofold change (log_2_ = 1) and *p *value < 0.05 (− log_10_ = 1.301). The abundance of proteins belonging to the large subunit (mtLSU, blue) of the mitoribosome are strongly reduced whilst the small subunit (mtSSU, aqua) is unchanged. **b** Topographical mapping of the protein fold-changes onto the mitoribosome structure (PDB: 3J9M). The protein abundance of mitoribosomal large subunit is reduced (mtLSU, blue), whereas the small subunit remains largely preserved. *ND* not detected. **c** C. Log2-transformed LFQ values from quantitative proteomics data comparing MRPL50 protein abundance between Twin 2 and Controls (*N* = 2) in triplicate. Mean ± 95% confidence interval is depicted. Statistical significance was determined using a two-tailed unpaired *t*-test. ****p < 0.0001. **d** Relative Complex Abundance (RCA) of OXPHOS complexes for *MRPL50* Twin 2 and *MRPL39* (Disease Control, DC) patients. The mean ratio of each protein within a complex is calculated, and a ratio paired *t*-test was performed to determine significance of each complex in the patient compared to control

Given *MRPL50* encodes a subunit of the mitochondrial ribosome, the proteomes of patient and control fibroblasts were assessed via quantitative proteomic analysis. A total of 4311 proteins (quantified from ≥ 2 peptides and representing ≥ 5% sequence coverage) of which 575 are annotated as mitochondrial (Rath et al. 2021) were quantified in fibroblasts of Twin 2 as well as two normal controls. One disease-affected control cell line derived from a patient with likely pathogenic variants in *MRPL39* (ClinVar Accession IDs VCV001676672.1 and VCV001676674.2) and presenting with severe paediatric mitochondrial disease were also used for comparison. The p.(Val112Asp) peptide was not detected in the MRPL50 patient (Supplementary File S2), likely due to the turnover of variant MRPL50 protein, whilst the wild-type peptide containing a Valine at position 112 was readily quantified in controls. To confirm this loss of protein was not due to cryptic changes to *MRPL50* splicing or gene expression, we performed RT-PCR and qRT-PCR which demonstrated normal splicing and retained mRNA expression (Supplementary Fig. S2). Consistent with a predicted effect on protein stability, we identified an 80.4% reduction in abundance of MRPL50 protein in patient fibroblasts relative to controls and reduced levels of most detected proteins previously shown to be associated with the large subunit of the mitochondrial ribosome (mtLSU), whilst proteins found associated with the small subunit (mtSSU) were unchanged relative to controls (Fig. 2a), suggesting destabilisation of the mtLSU. Additionally, Log2-transformed LFQ intensity values from quantitative proteomics data demonstrated a significant reduction in abundance of MRPL50 protein when compared with controls (Fig. 2c). Topographical mapping of mitoribosomal protein abundance onto the 3D structure of the complex also showed a global decrease in abundance of proteins of the large subunit, whereas those of the small subunit were predominantly unchanged (Fig. 2b). The mitochondrial ribosome is responsible for the translation of mtDNA-encoded subunits of the OXPHOS machinery, and patient fibroblasts had a mild but significant decrease in the abundance of mitochondrial complex I (Fig. 2d).

**Fig. 3:**
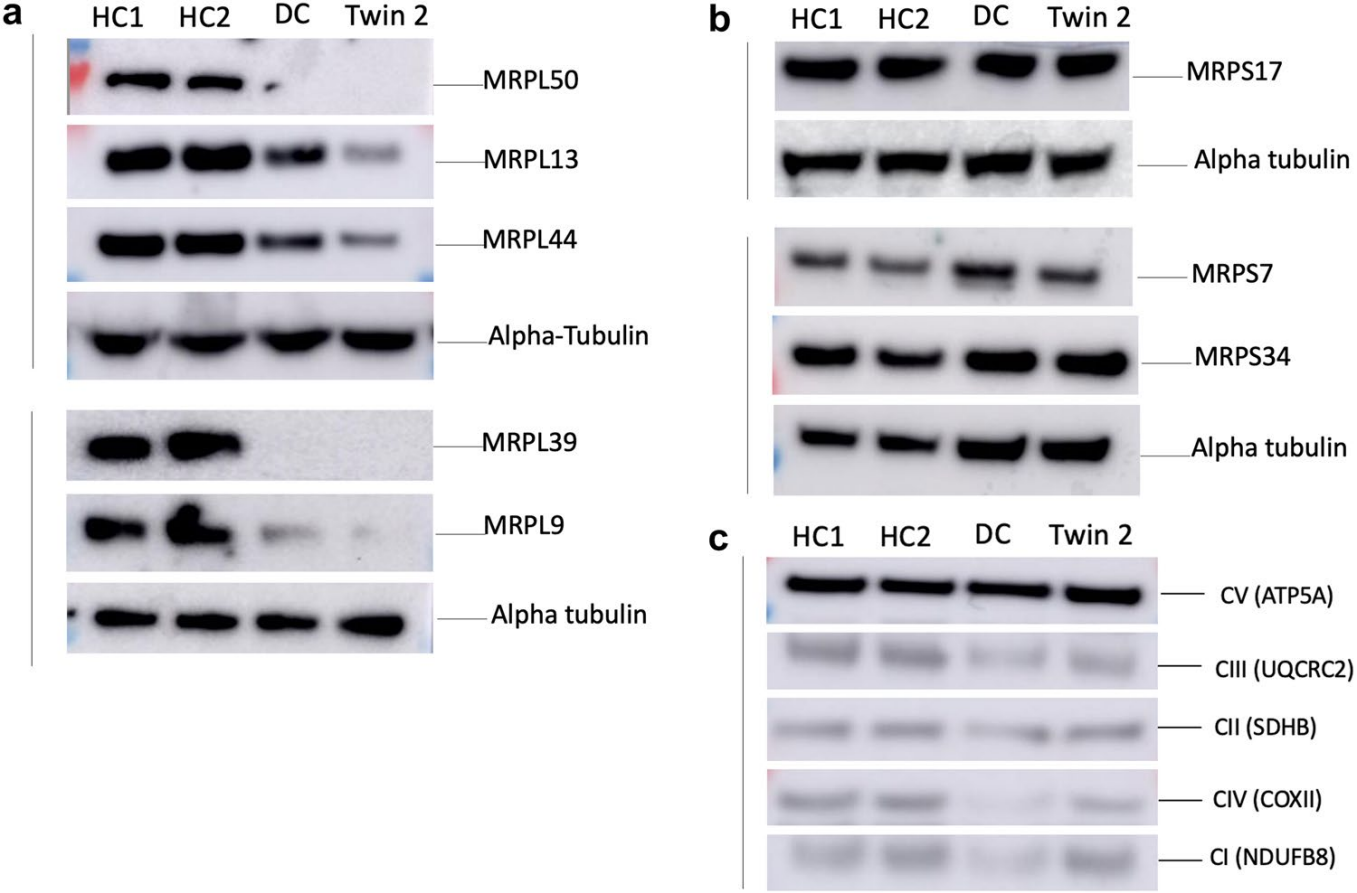
MRPL50 variant is associated with reduced protein levels of multiple large mitoribosomal subunits reflecting destabilisation of the mitoribosome. Whole cell lysates from fibroblasts of two healthy controls (HC1 and HC2), a disease control with variants in *MRPL39* (DC) and Twin 2 were analysed by western blot. **a** Western blot using antibodies against proteins of the large subunit of mitochondrial ribosome showed reduced protein levels of MRPL50, MRPL13, MRPL44, MRPL39 and MRPL9 in Twin 2 and DC fibroblast cells relative to healthy control individuals, **b** whereas the expression levels in proteins of small subunit of mitochondrial ribosome including MRPS17, MRPS7 and MRPS34 were unchanged. **c** Western blot using antibodies against components of the oxidative phosphorylation machinery revealed no detectable deficiency in the OXPHOS proteins in MRPL50 patient cells compared with the controls. Vertical lines indicate individual membranes that have been probed with each of the antibodies shown in that set including a loading control for each

Consistent with quantitative proteomics, western blot analysis of patient fibroblasts also showed decreased levels of MRPL50, MRPL13, MRPL44, MRPL39 and MRPL9 whilst the MRPS17, MRPS7 and MRPS34 levels were unchanged relative to control individuals (Fig. 3a, b). There was no clear deficiency in the OXPHOS proteins tested, likely due to the reduced sensitivity and dynamic range of western blotting compared to proteomics, where only a mild complex I deficiency was observed (Fig. 3c). Although proteomics detected a significant but mild complex I deficiency, the individual subunits that were significantly reduced were NDUFB11, NDUFB5, NDUFC2, NDUFV3 and NDUFS5, whilst the remaining subunits, including NDUFB8 which is detected by the OXPHOS cocktail antibody, were not significantly decreased compared to controls.

Interestingly, the disease controls (DC with likely pathogenic variants in *MRPL39*, ClinVar Accession IDs VCV001676672.1 and VCV001676674.2), who died in infancy from a cardioencephalomyopathy, had much more severe biochemical defect with a more pronounced OXPHOS deficiency in fibroblasts, indicating possible correlation between disease phenotype and severity of the biochemical defect.

### Abnormal ovarian and testicular structure/function after mRpL50 knockdown and knockout in Drosophila disease models

**Fig. 4:**
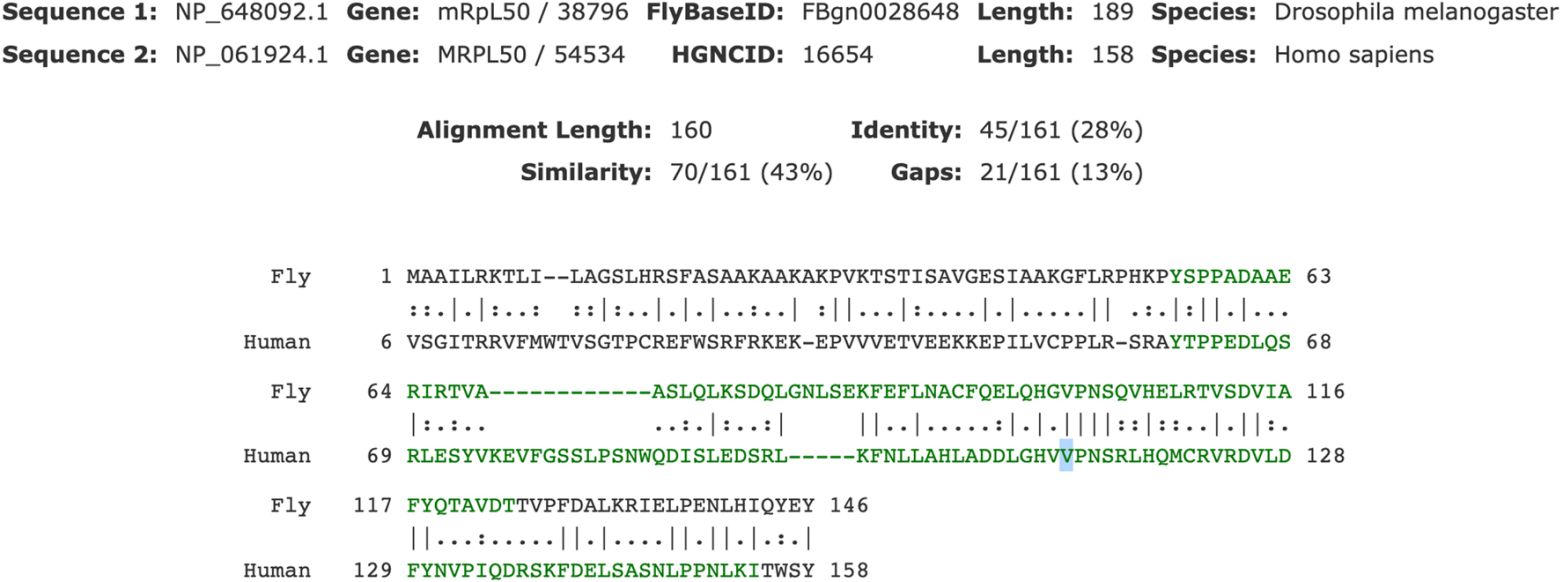
Protein alignment of human MRPL50 and its orthologue, mRpL50, in *Drosophila*. mRpL50 shows 28% identity (lines) and 43% similarity (double dots) to the human MRPL50 protein. It shows very high conservation around the variant and the residue (highlighted) is identical in fly

The analysis of patient fibroblasts demonstrated a clear biochemical defect related to MRPL50 deficiency but to prove causality, a link between the gene and the clinical phenotype was sought. To support causality therefore, we generated tissue-specific mRpL50-RNAi knockdown and CRISPR-Cas9-mediated mRpL50-knockout *Drosophila* models. Given proteomic data demonstrated > 80% reduction of MRPL50 protein and absence of the variant peptide, these fly models recapitulated the human scenario with reduction/loss of mRpL50 rather than stable expression of variant protein. We analysed the impact of mRpL50 deficiency and assessed ovarian structure and function. The orthologue of human *MRPL50* in *Drosophila*, *mRpL50*, encodes a protein with 28% identity and 43% similarity to the human MRPL50 protein (www.flybase.org). As indicated by the alignment in Fig. 4, it shows very high conservation around the variant and the affected residue is identical in fly.

**Fig. 5:**
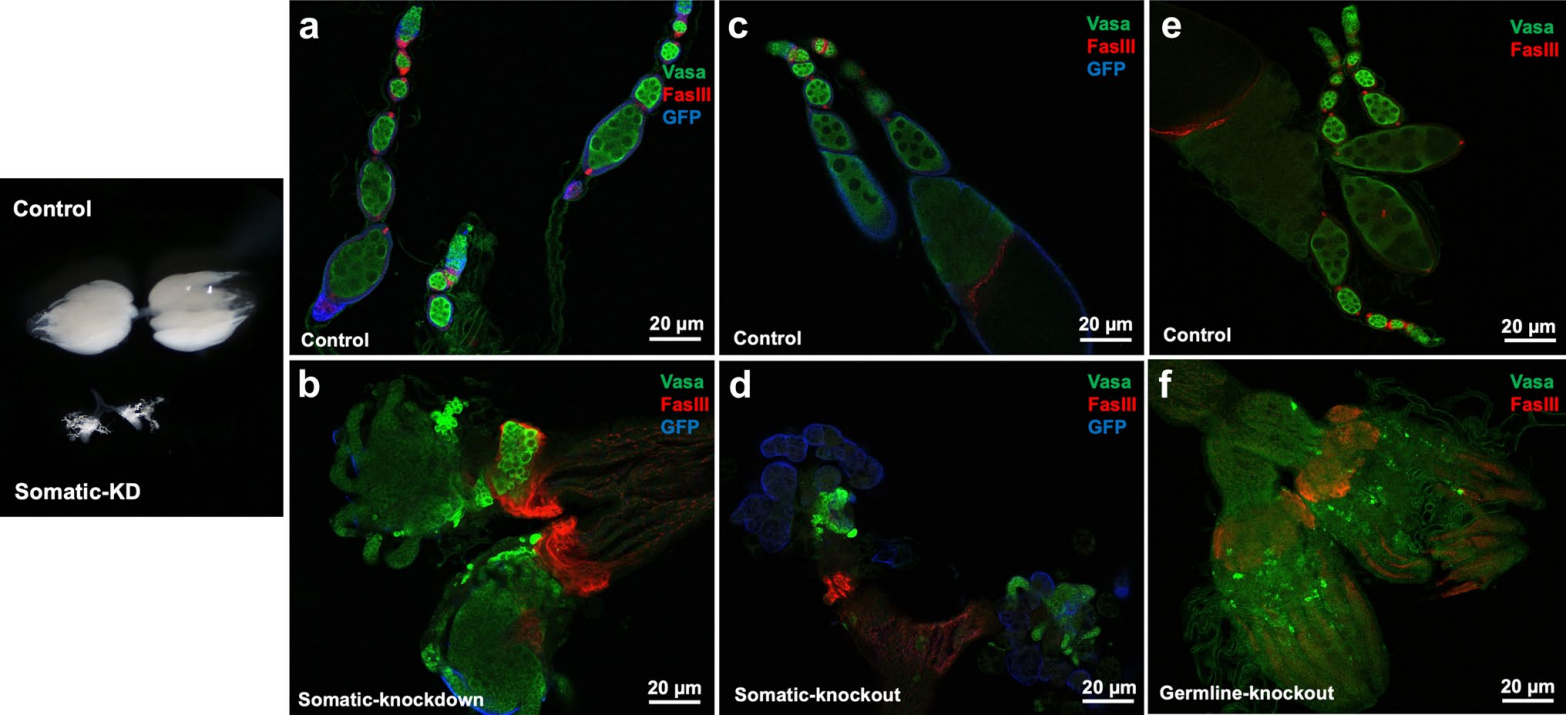
mRpL50 is required for ovarian development and function in *Drosophila*. Representative confocal images of ovarioles from controls (**a**, **c**, **e**) and somatic knockdown (**b**), somatic knockout (**d**) and germline knockout (**f**) females. Vasa protein (green, germ cells), FasIII (red, somatic follicle cells), GFP (blue, indicating domain of Tj-GAL4 expression in somatic support cells). Black and white image on the left show intact dissected ovaries of control and somatic-KD flies

**Fig. 6:**
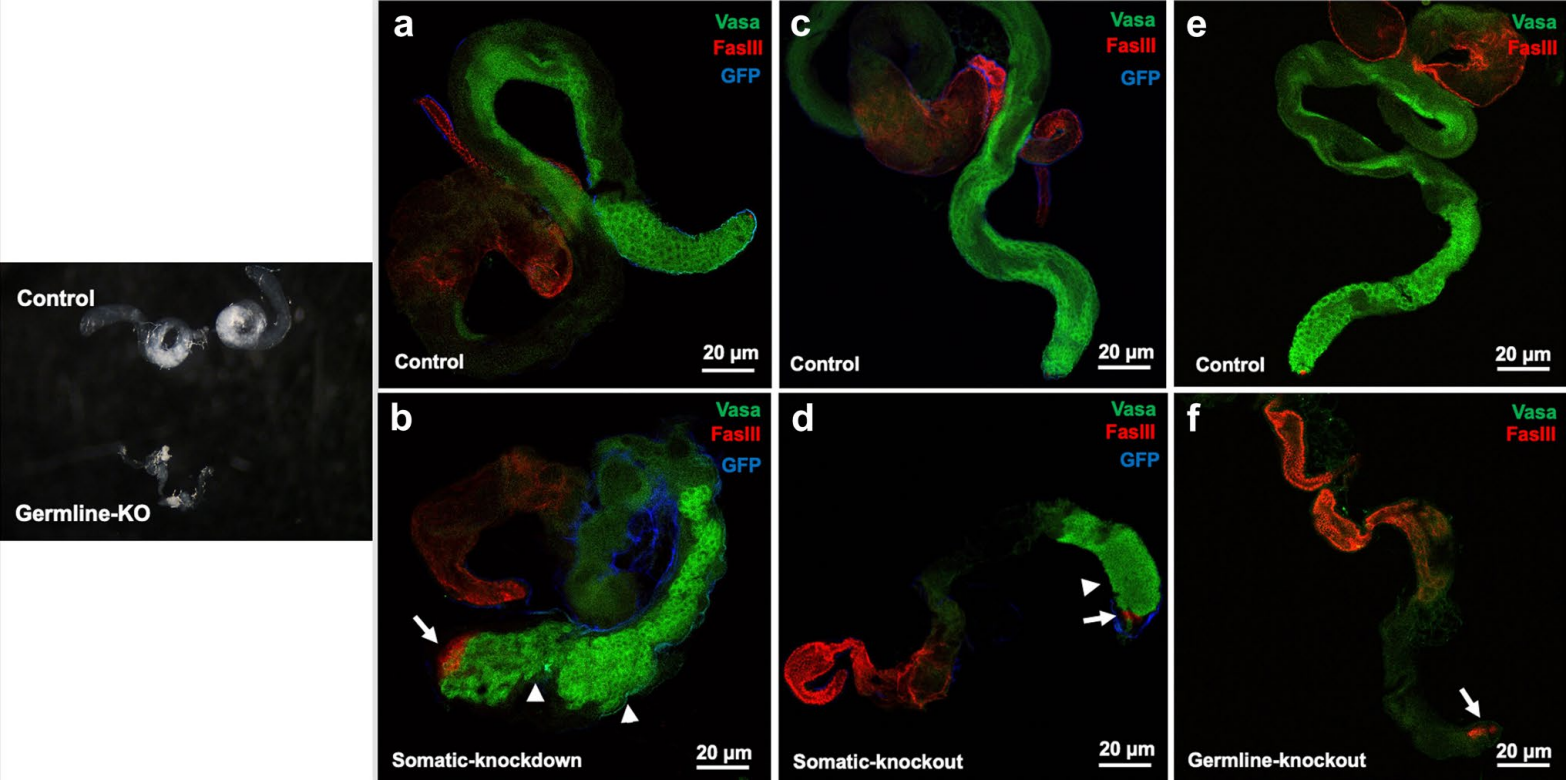
mRpL50 deficiency leads to abnormal structure and function of testis in Drosophila. Representative confocal images of testes from controls (**a**, **c**, **e**) and somatic knockdown (**b**), somatic knockout (**d**) and germline knockout (**f**) males. Vasa protein (green, germ cells), FasIII (red, somatic hub and terminal epithelial cells), GFP (blue, indicating Tj-GAL4 expression in somatic support cells). Arrows show the expansion in the size of stem cell niche (hub) and arrowheads show over-proliferating dysregulated early germ cells. Black and white image on the left show intact dissected testes of control and germline-KO flies

*Drosophila* strains with somatic- and germ-cell-specific double RNAi knockdown were analysed by gonadal dissection and immunostaining. Knockdown in somatic tissues revealed a small, undeveloped and abnormal ovarian structure with failure to develop differentiated ovarian egg chambers and defects in encapsulation of germ cells into ovarian cysts in somatic cell-derived knockdown flies compared with control groups in which ovarioles showed strings of developing egg chambers (Fig. 5a, b). The knockdown ovaries were associated with accumulation of undifferentiated germ cells (Fig. 5b). We concurrently analysed testicular structure and found a similar phenotype with small and undeveloped testes with over-proliferating dysregulated early germ cells in somatic cell-derived knockdown flies. Moreover, there were an expansion in the apparent size of the hub (stem cell niche), associated with transdifferentiation of hub cells into the somatic stem cell lineage with retention of hub markers, in somatic cell-derived knockdown flies compared with normal testicular structure in control groups (Fig. 6e, f). In contrast, germline-specific knockdown of mRpL50 in *Drosophila* did not show any obvious phenotype in ovaries or testes (data not shown).

There is evidence to suggest that germ cell-specific knockdown in *Drosophila* is inefficient in female germ cells due to the expression of piRNAs derived from the Hsp70 locus that interfere with expression from pUAST-derived vectors (DeLuca and Spradling 2018). This fact, with the lack of ovarian phenotype observed in the germ-cell-specific knockdown flies, prompted us to investigate the impact of CRISPR-Cas9-mediated knockout of mRpL50 (KO).

Supporting knockdown results, immunostaining of dissected gonads from somatic cell-knockout flies (Figs. 5c, d, 6c, d) showed a similar abnormal ovarian and testicular phenotype, although in both cases the accumulation of undifferentiated germ cells was much more apparent. Both the testes somatic cell knockdown and knockout (Fig. 6b, d) exhibited an expansion in the number of spermatogonia with limited differentiation into spermatocytes. In contrast to the knockdown results, however, germline-knockout flies (Figs. 5e, f, 6a, b) also showed abnormal ovarian and testicular structure. Germline knockout ovaries appeared to be essentially agametic, with very few or no germ cells present. Interestingly, germ cells were also lost from the germline-knockout testes and the FasIII staining indicated an increase in the apparent area of the stem cell niche (Fig. 6a, b), as is commonly observed in mutants that lose germline stem cells. After incubating transgenic flies together for 4 + days at 29 degrees, no eggs were observed, confirming impaired fertility.

## Discussion

### The MRPL50 missense variant disrupts protein stability

Our data indicate that the *MRPL50* missense variant results in reduced abundance of MRPL50 protein, indicating the pathogenic mechanism of this variant is likely an impact on protein stability rather than leading to a hypomorphic protein. Our data indicate that *MRPL50* may be a non-essential gene. Consistent with this notion, loss-of-function variants are observed at the expected frequency in gnomAD in contrast to known essential genes for which loss-of-function variants are observed at below the expected frequency (Karczewski et al. 2020). Although missense variants are more frequently reported in mitoribosome genes, bi-allelic predicted loss-of-function variants have been described in other mitochondrial ribosome subunits with a similar effect on mitoribosome stability (Lake et al. 2017b). It remains possible, however, that the low level of residual expression of variant protein in these cases enables survival beyond birth.

### Biallelic missense variants in *MRPL50* are a new cause of syndromic POI manifesting as Perrault syndrome

Here we present data to support MRPL50 deficiency as a novel cause of a mitochondrial disorder, manifesting with premature ovarian insufficiency, sensorineural hearing loss and chronic kidney disease in dizygotic twin sisters. This is the first report of MRPL50 deficiency, and our proteomic analysis in patient fibroblasts and *Drosophila* disease modelling consolidates the important role of MRPL50 and the mitochondrial ribosome in supporting ovarian function.

POI is a heterogeneous disorder that can be associated with significant co-morbidity, which depends on the underlying genetic disorder including leukoencephalopathy, ataxia telangiectasia, cardiovascular disease, and Perrault syndrome. Perrault syndrome (MIM: 233400) is a heterogeneous autosomal recessive disorder, characterised by ovarian dysfunction in females and sensorineural hearing loss in both males and females (Pallister 1979). There is phenotype variability in the manifestation of disease in patients with Perrault syndrome. Ovarian dysfunction can vary from ovarian dysgenesis with poorly developed streak ovaries and primary amenorrhea to premature ovarian insufficiency manifesting as secondary amenorrhea. Sensorineural hearing loss in the affected individuals described in the literature also varies from bilateral mild to moderate post-lingual delayed onset to profound pre-lingual onset that occurs before learning language. The ovarian phenotype in our patients was early-onset POI, presenting as secondary amenorrhea with only 3 menstrual cycles in one of the twins (Twin 1) and primary amenorrhea in the other (Twin 2). Audiometry showed bilateral normal-profound steeply sloping sensorineural hearing loss with delayed onset around 24 years of age in both twins. In some Perrault syndrome cases, the presence of neurological manifestations has been noted, such as neuropathies, intellectual disability, and ataxia (Newman et al. 2018). Beyond the premature ovarian insufficiency and hearing loss, our patients had left ventricular hypertrophy that could be secondary to chronic kidney disease as part of their clinical phenotype, showing the multi-organ nature of this disorder. Furthermore, these twins might be at heightened risk of neurological manifestations later in life.

Our *Drosophila* modelling demonstrated the link between *MRPL50* and ovarian pathology, but the causal link between *MRPL50* disruption and other aspects of the patient phenotype (hearing loss, kidney and heart dysfunction) has not directly been validated. Mitochondrial function, however, is known to be necessary for function of these related organs as they have high energy demand and can be susceptible to oxidative damage (Kirkman et al. 2008). We have conclusively demonstrated disrupted mitochondrial ribosome stability in patient fibroblasts and this is an established cause of these clinical features. For example, disruption to *MRPS7* is known to result in POI, hearing loss and kidney failure (Kline et al. 2022; Menezes et al. 2015). Similarly, variants in *MRPL44* are known to cause cardiomyopathy (Carroll et al. 2013; Friederich et al. 2021). Further cases of *MRPL50* deficiency described in the literature are likely to confirm the clinical spectrum of this disorder. Our data add to the growing evidence that the mitochondrial ribosome is integral for hearing and ovarian, kidney and heart function.

**Fig. 7:**
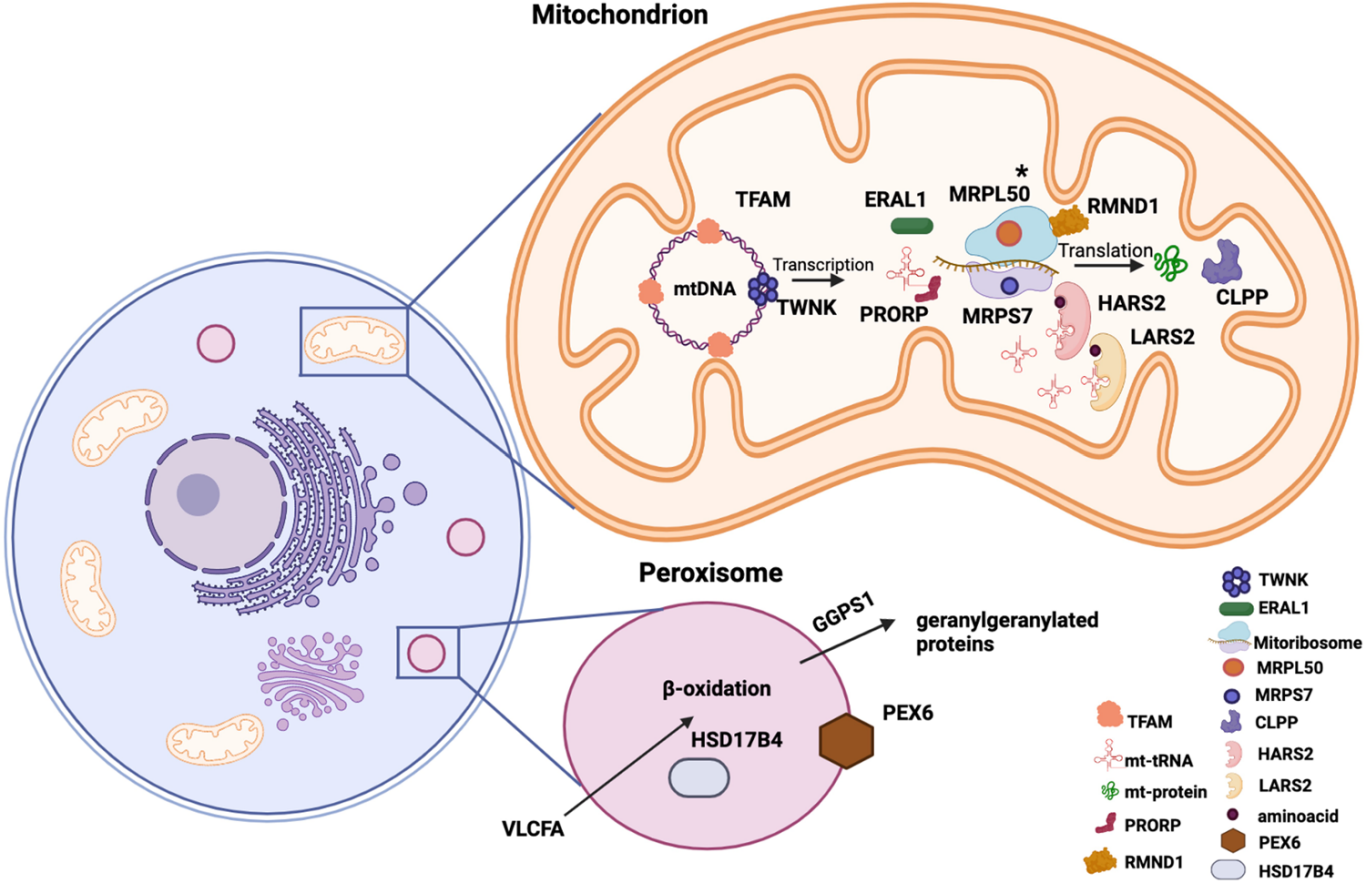
A Schematic diagram illustrates proteins implicated in Perrault syndrome. Most of these proteins have a role in maintaining mitochondrial protein homeostasis and involved in mtDNA replication or translation. TWNK contributes to mtDNA replication by unwinding the double strands of mtDNA, then making them accessible to the mitochondrial transcription (POLRMT) and replication (POLG) polymerases. TFAM is a mitochondrial transcription factor required for mtDNA transcription. ERAL1 is a mitochondrial GTPase and enables mt-protein translation by playing a role in the assembly of the small 28S subunit of the mitochondrial ribosome. HARS2 and LARS2 are mitochondrial histidyl and leucyl-tRNA synthetases, which charge mitochondrial tRNAs with their cognate amino acids. CLPP is a proteolytic subunit of mitochondrial ATP-dependent Clp protease complex, which is an enzyme responsible for the degradation of unfolded proteins and affects mitochondrial function. PRORP is a catalytic ribonuclease component of mitochondrial ribonuclease P and it cuts tRNA molecules in their 5′-ends, which is an essential step to generating functional mitochondrial tRNA molecules. RMND1 contributes to assembly of the mitochondrial ribosome and supports translation of mtDNA-encoded polypeptides. MRPL50 and MRPS7 are components of the mitochondrial large and small ribosomal subunits respectively, required for synthesis of mtDNA-encoded proteins. MRPL50 described in our study is highlighted by an asterisk. The other organelle involved in Perrault syndrome pathogenesis is the peroxisome. The process of oxidizing very long-chain fatty acids (VLCFA) takes place in the peroxisome. HSD17B4 is an enzyme that is involved in this beta-oxidation pathway. A peroxisomal biogenesis factor, PEX6, is required for the import of proteins into peroxisomes through the peroxisomal membrane. As a geranylgeranyl diphosphate synthase, GGPS1 catalyses the synthesis of Geranylgeranyl Pyrophosphate (GGPP) required for protein prenylation. [Diagram is adapted from Tucker et al. (2020)]. Diagram is created with using BioRender

A growing number of bi-allelic variants in several genes identified by next-generation sequencing have been reported to be causative for Perrault syndrome; however, the molecular diagnosis has not been established in approximately 60% of patients identified with Perrault syndrome (Newman et al. 2018). These genes, most involved in maintaining mitochondrial protein homeostasis, include *CLPP*, *HARS2*, *LARS2*, *ERAL1*, *TWNK*, *TFAM*, *RMND1* and *PRORP* (Fig. 7). Causative variants in *CLPP* are most often missense variants with a possible mutational hotspot identified in the functional domain at residue p.Cys147. These variants in CLPP have been discovered to cause Perrault syndrome associated with hearing loss and ovarian dysfunction as well as neurologic features (Lerat et al. 2016; Tucker et al. 2020). Variants in *HARS2* and *LARS2* are associated with Perrault syndrome manifesting with a wide range of neurological symptoms, variable progression of hearing loss and POI (Carminho-Rodrigues et al. 2020; Kosaki et al. 2018; Pierce et al. 2013; Souissi et al. 2021). A homozygous missense variant in *ERAL1* was associated with disrupted mitochondrial function, sensorineural hearing loss and ovarian dysgenesis (Chatzispyrou et al. 2017). *TWNK* is also believed to cause Perrault syndrome when harbouring deleterious variants (Morino et al. 2014). Furthermore, homozygous missense variants in *TFAM* have been reported in POI patients presenting with primary amenorrhea, atrophic ovaries, seizures, intellectual disability as well as clinical features of Perrault syndrome (Tucker et al. 2020; Ullah et al. 2021). Two compound heterozygous missense variants in *RMND1* are implicated with ovarian dysfunction, Perrault syndrome and chronic kidney disease (Oziębło et al. 2020). Perrault syndrome presenting with hearing loss and POI has also been reported as a result of a homozygous variant in *PRORP* (Hochberg et al. 2021).

In addition to well-established Perrault syndrome genes involved in mitochondrial function, recent studies have described causative variants in non-mitochondrial genes including *HSD17B4*, *GGPS1* and *PEX6* in patients presenting with clinical features overlapping with Perrault syndrome (Fig. 7) (Tucker et al. 2020). In the current study, the cumulative evidence of proteomic analysis and animal modelling in *Drosophila* supports the pathogenicity of homozygous missense variants in *MRPL50* being a novel cause of syndromic POI. Although our data support the association of *MRPL50* with the clinical phenotype of syndromic POI, independent families with different bi-allelic variants in *MRPL50* and overlapping phenotype are required to confirm this gene–disease relationship. The twins of this study additionally presented with chronic kidney disease and left ventricular hypertrophy. Further cases may elucidate whether these patients have primary cardiac disease [as is the case for patients with variants in the related *MRPS14*, *MRPS22*, *MRPL3*, and *MRPL44* genes (Carroll et al. 2013; Distelmaier et al. 2015b; Friederich et al. 2021; Galmiche et al. 2011b; Jackson et al. 2019b; Smits et al. 2011b)] or whether this cardiomyopathy is secondary to chronic kidney disease, for example, if patients present with cardiac disease in the absence of, or prior to the development of, kidney disease. Expanded understanding of genotypic cause and associated phenotypic insights will improve future patient diagnoses and management.

### Mitochondrial function in ovarian development and mitoribosome-related POI

Mitochondria are maternally inherited organelles controlled by the mitochondrial and nuclear genomes. They generate adenosine triphosphate (ATP) via the process of OXPHOS as the cellular powerhouses (Pfanner et al. 2019). Most mitochondrial proteins, such as the multi-subunit OXPHOS complexes (complex I to complex V), required for normal function of mitochondria, are encoded by the nuclear genome (Tiosano et al. 2019). Mitochondria are directly involved in a wide range of female reproductive processes including oocyte maturation during oogenesis, steroid hormone production, fertilization and different stages of embryonic development (May‐Panloup et al. 2007). During oocyte maturation, mitochondria localise and accumulate around the forming meiotic spindles in the ooplasm to meet the energy demands of germinal vesicle breakdown and assembly of microtubules (Coticchio et al. 2015; Yu et al. 2010). Disrupted mitochondrial function and impaired OXPHOS in mouse oocytes cause defective meiotic spindle formation and oocyte maturation (Zhang et al. 2006). Mitochondrial biogenesis plays a central role in the determination of the size of the follicular pool during intrauterine life (Aiken et al. 2016). Several studies in mouse models have indicated the importance of mitochondria in follicular atresia due to their roles in cell survival and apoptosis. Mitochondrial dysfunction causes reduced OXPHOS and increased ROS levels, which induce apoptosis. Subsequently, increased apoptosis rate in oocytes and surrounding follicular cells leads to depletion of ovarian follicles (Hussein 2005; May-Panloup et al. 2016). Moreover, many studies have shown that supra-physiological levels of ROS can cause cellular oxidative stress and adversely affect the quality of developing oogonia (Kumar et al. 2012; Prasad et al. 2016; Venkatesh et al. 2010). Oxidative stress can cause ovarian dysfunction and female reproductive disorders, such as polycystic ovary syndrome (PCOS), POI, endometriosis and infertility (Lu et al. 2018). Our study showed that disruption of mitochondrial ribosome biogenesis through MRPL50 deficiency, results in defective complex I biogenesis, disturbs ovarian development and function. This is highlighted by MRPL50-deficient *Drosophila* models showing abnormal ovarian structure and having subsequent infertility.

Identifying *MRPL50* as a mitochondrial disease gene associated with POI adds to growing evidence that the mitochondrial ribosome is critical for ovarian function and fertility. Homozygous missense variants in *MRPS22*, a component of the mitochondrial small subunit, have been previously reported in non-syndromic POI patients and animal models were used for the verification of the variants in human POI pathogenesis (Chen et al. 2018a). Two siblings with a homozygous variant in *MRPS7* (MIM: 611974) experienced liver and renal disease, which progressed to early death in one sister. The other sister with a milder disease course, however, experienced failure of pubertal development and hypogonadism indicating the requirement of MRPS7 for reproductive function. Our recent study also described two heterozygous variants in *MRPS7* in an individual diagnosed with POI experiencing secondary amenorrhea and sensorineural hearing loss (Kline et al. 2022). Altogether these studies accentuate the varied phenotypic features of disorders associated with impaired mitoribosomal function and the repeated involvement of ovarian dysfunction.

### *Drosophila* models validating new ovarian POI genes

Our study has demonstrated that deficiency of the MRPL50 orthologue in *Drosophila* leads to stunted ovarian development and small-sized ovaries devoid of germ cells or exhibiting a failure in normal germ cell differentiation. Interestingly, a similar phenotype was observed in male mRpL50-deficient flies that had small and undeveloped testes. This suggests that the role of *MRPL50* may not be limited to supporting female fertility but also male fertility. *MRPL50* could therefore be considered a candidate gene underlying male infertility, consistent with many known POI genes that can cause infertility of both sexes (e.g. *STAG3*, *ZSWIM7*, etc.). Our study demonstrates the utility of *Drosophila* for modelling infertility. For example, whilst the complete deficiency of Mrps22 led to mouse embryonic lethality (Chen et al. 2018a), germ cell-specific deficiency in *Drosophila* confirmed the requirement of this gene for ovarian function (Chen et al. 2018a). Our data suggest that mRpL50-deficient *Drosophila* recapitulate the reproductive phenotype manifested in patients harbouring *MRPL50* variants, supporting the effect of *MRPL50* disruption in the pathogenesis of human disease.

### Proteomic analysis can complement genomic analysis

Whole-exome sequencing is increasingly being used to identify the genetic cause of rare diseases. One of the challenges of this high-throughput gene sequencing is distinguishing between benign and pathogenic variants. Our study demonstrates that coupling of quantitative proteomics data with exome sequencing data, can help confirm variant causation. In silico predictions suggested that the *MRPL50* p.(Val112Asp) variant was likely deleterious, whilst proteomic data could confirm the reduction in MRPL50 abundance as well as most other proteins found within the large subunit of the mitoribosome (mtLSU). Quantitative proteomics also detected a significant reduction in the abundance of complex I subunits, consistent with a defect in the synthesis of mtDNA-encoded proteins. The significant reduction of only complex I in this patient might be due to the higher number of mtDNA-encoded subunits in complex I compared to other OXPHOS complexes; however, we note that the level of complex IV subunits quantified in the patient is trending down, although not reaching statistical significance. A combined OXPHOS defect is often indicative of mtDNA translation defects, as seen in the control disease-affected patient (Fig. 2c) and in published reports (Gardeitchik et al. 2018; Lake et al. 2017a).

Our data also indicate the potential for quantitative proteomic analysis to provide insight into disease severity. A mild OXPHOS deficiency was detected in fibroblasts from the patients with MPRL50 deficiency and a relatively mild disease course, surviving to adulthood, in contrast to a severe deficiency in OXPHOS complexes correlating with severe disease in the disease control who died in infancy from a cardioencephalomyopathy (Fig. 2d). This indicates a possible correlation between phenotype severity and biochemical defects, although more patients must be analysed via proteomics to confirm this association. On the other hand, mitochondrial defects are known to have tissue-specificity, and it remains possible that the mild complex I deficiency detected in patient fibroblasts could be more pronounced if kidney or ovarian samples were available for analysis. These findings highlight the significance of proteomic analysis in providing functional data to confirm genomic diagnosis. There is a need to analyse further undiagnosed patients/families to understand the spectrum of disease presentation due to variants in the *MRPL50* gene.

In conclusion, here we provide the first evidence of bi-allelic variants in *MRPL50* as a cause of mitochondrial disease. The c.335T > A; p.(Val112Asp) variant identified in twin sisters from a third-degree consanguineous marriage is likely responsible for autosomal recessive inheritance of syndromic POI, associated with hearing loss, chronic kidney disease and left ventricular hypertrophy. We showed evidence for *MRPL50* variant causation using quantitative proteomic analysis in patient fibroblasts and generation of *Drosophila* disease models. Our data highlight the critical role of mitochondria in human ovarian function.

## Supplementary Information

Below is the link to the electronic supplementary material.

Supplementary file 1 (XLSX 129 kb)

Supplementary file 2 (XLSX 1268 kb)

Supplementary file 3 (PDF 341 kb)

## Data Availability

Some or all datasets generated during and/or analysed during the current study are not publicly available but are available from the corresponding author on reasonable request. MRPL50 variant is submitted to ClinVar (https://www.ncbi.nlm.nih.gov/clinvar/ submitted on 20 December 2022) Submission ID: SUB12455331.
