# Supplementary material for: Deficiency of the mitochondrial ribosomal subunit, MRPL50, causes autosomal recessive syndromic premature ovarian insufficiency
Source: Hum Genet. 2023 May 6;142(7):879–907. doi: 10.1007/s00439-023-02563-z (PMC10329598; doi:10.1007/s00439-023-02563-z)
Supplement: Supplementary file 3 — (PDF 341 kb) [file 439_2023_2563_MOESM3_ESM.pdf]

Twin 1

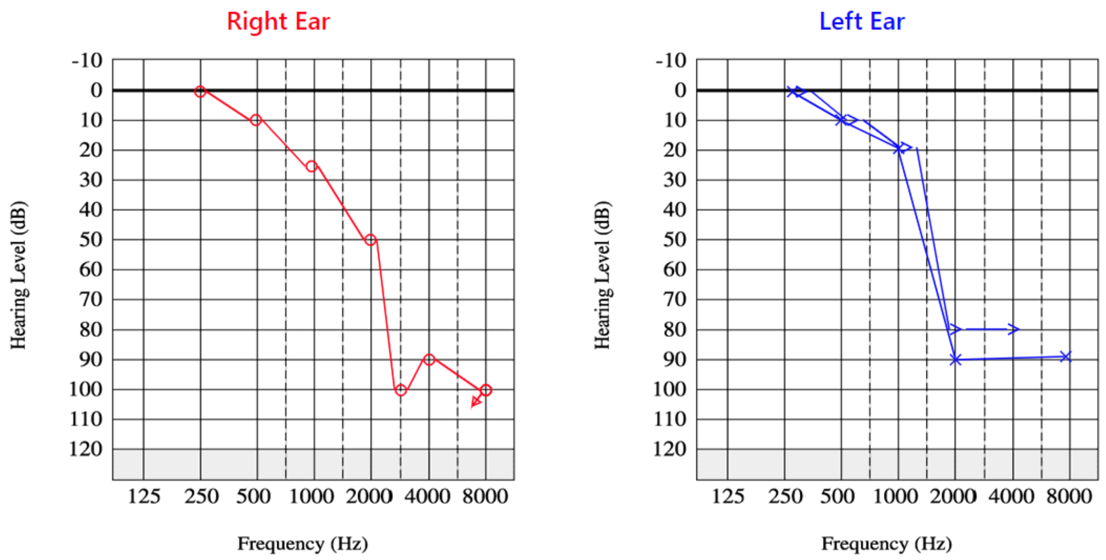

Twin 2

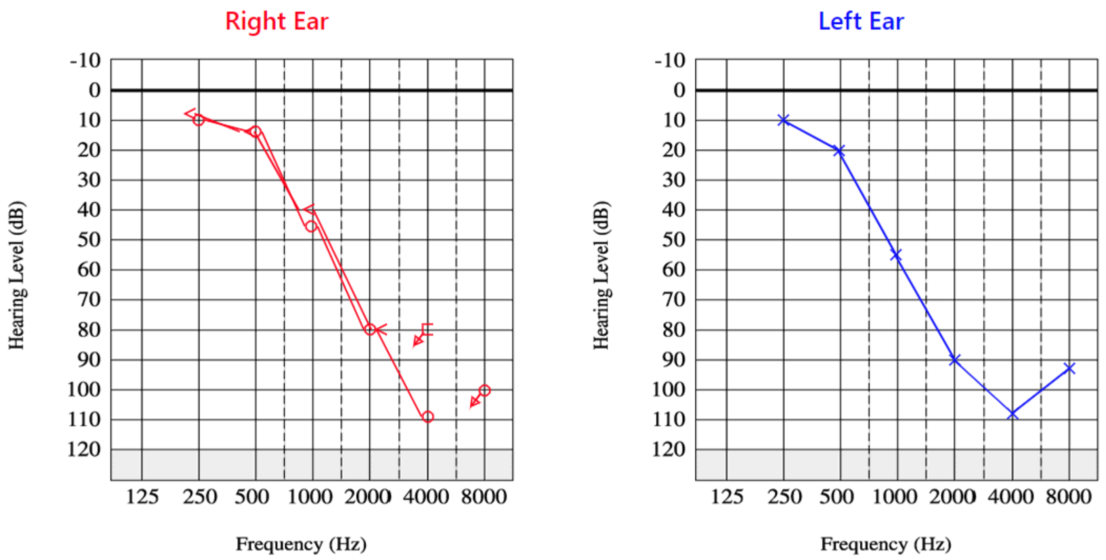

Supplementary Figure S1: Pure-tone audiometry results for Twin 1 (upper) and Twin 2 (lower). The Y-axis (vertical) shows the intensity of the sound, in decibels (dB HL). The X-axis (horizontal) shows the frequency of sound in Hz. Crosses indicate air-conduction thresholds for the left ear, and circles indicate air-conduction thresholds for the right ear. The symbol › is used to show bone conduction thresholds. An arrow symbol indicates no response to sound at that frequency.

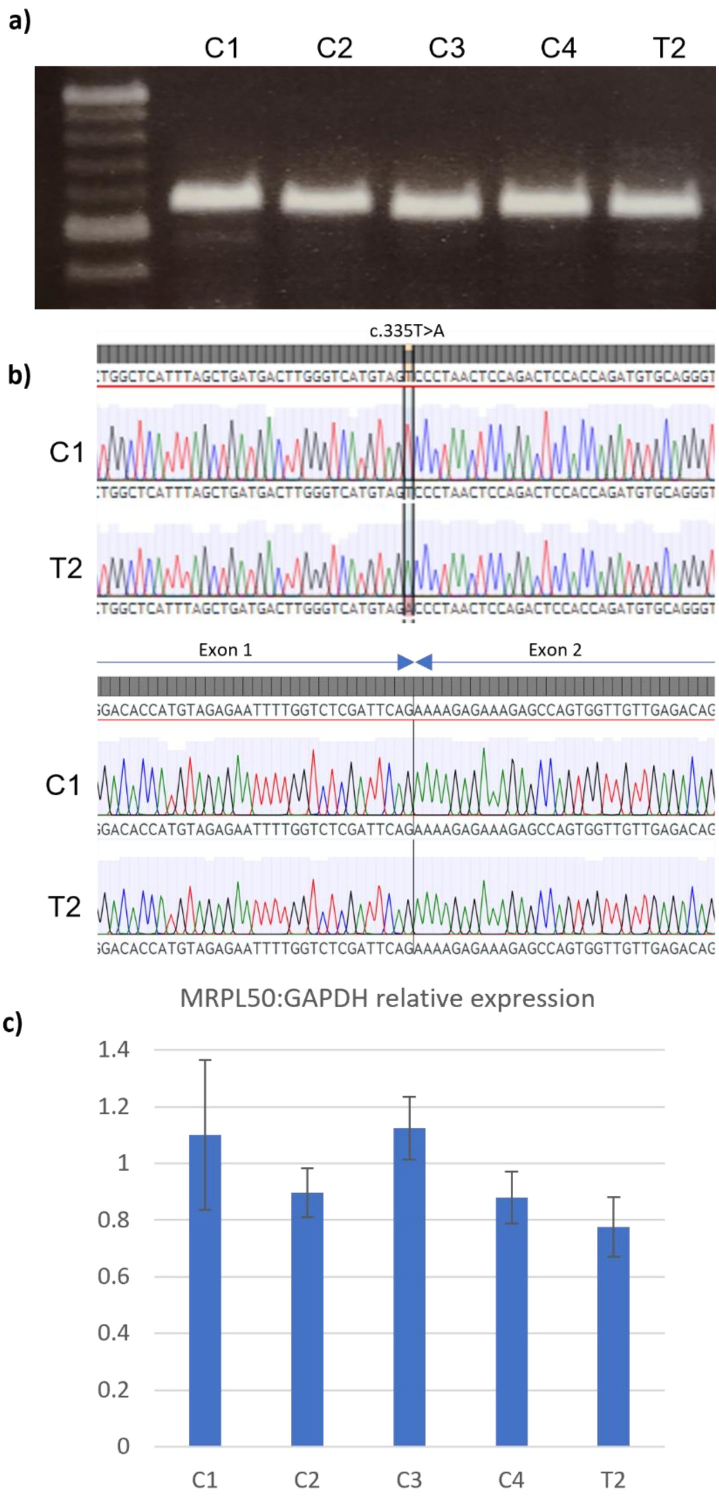

Supplementary Figure S2. The MRPL50 variant does not have a significant impact on MRPL50 transcript expression. a) Full-length RT-PCR of control (C1-C4) and patient (T2) lymphoblast cDNA demonstrates no discernable disruption to MRPL50 splicing b) Chromatograms show the homozygous missense variant in patient cDNA (upper) and normal MRPL50 exon-exon boundary indicating normal splicing (lower). c) qRT-PCR demonstrates that relative MRPL50 expression in T2 does not differ significantly from that in controls (C1-C4). Bars represent the mean of three independent experiments, each performed in triplicate. Error bars: ±1 SEM.
